# 15-Fold increase in solar thermoelectric generator performance through femtosecond-laser spectral engineering and thermal management

**DOI:** 10.1038/s41377-025-01916-9

**Published:** 2025-08-12

**Authors:** Tianshu Xu, Ran Wei, Subhash C. Singh, Chunlei Guo

**Affiliations:** The Institute of Optics, University of Rochester, Rochester, NY 14627 USA

**Keywords:** Solar energy and photovoltaic technology, Laser material processing, Ultrafast lasers, Nanoparticles

## Abstract

Solar thermoelectric generators (STEGs) have recently gained increasing attention. However, their widespread adoption has been limited due to the lack of high-efficiency thermoelectric materials and compact heat sinks for effective heat dissipation. To address these issues, we develop a spectral engineering and thermal management strategy that significantly increases STEG power generation by 15 times with only a 25% increase in weight. At the hot side, we transform a regular tungsten (W) to a selective solar absorber (W-SSA) through a femtosecond (fs)-laser processing technique, which enhances the solar absorption while minimizing the IR emissivity, obtaining >80% absorption efficiency at elevated temperatures. We also design a greenhouse chamber for W-SSA and achieved >40% reduction in convective heat loss. At the cold side, we apply the fs laser processing to transform a regular aluminum (Al) to a super-high-capacity micro-structured heat dissipator (μ-dissipator), which improves the cold-side heat dissipation through both radiation and convection, achieving twice the cooling performance of a regular Al heat dissipator. These spectral engineering and thermal management increase the temperature difference across the STEG, resulting in a substantial increase in output power. The high-efficiency STEG can find a wide range of applications, such as wireless sensor networks, wearable electronics, and medical sensors.

## Abstract (web summary)

A spectral engineering and thermal management strategy is developed to significantly increase STEG power generation. It consists of a fs-laser-treated W-SSA, a greenhouse chamber, and a fs-laser-treated μ-dissipator.

## Graphical abstract

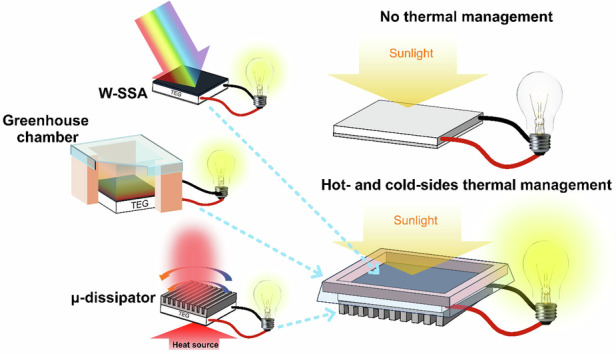

## Introduction

Solar energy is a main source of renewable energy. Solar photovoltaics (PV) and solar thermoelectric (TE) are the most widely used technologies^1–4^. Based on the Seebeck effect, TE materials will generate a voltage when subjected to a temperature difference (Δ*T*) across their ends. When these materials are positioned between a solar absorber and a heat dissipator to establish a Δ*T* and produce power, they are referred to as solar thermoelectric generators (STEGs)^1,4–7^. The STEG’s thermal-to-electrical conversion efficiency, ${\eta }_{{STEG}}$, is generally determined by a dimensionless figure-of-merit (*ZT*) of the TE material and the temperature difference ($\varDelta T$) between hot-side (${T}_{h}$) and cold-side temperatures (${T}_{c}$) across the device^1,4^. Unlike solar PVs that can only utilize a narrow band of sunlight near the bandgap of the semiconductor material, STEGs have solar absorbers with a much wider absorption band, so the photon energy of the entire solar spectrum can be utilized^3^. However, STEG’s ${\eta }_{{STEG}}$ is very low, usually below 1% without solar concentration due to a lack of high *ZT* TE material, which limits the output power and their commercial adoption. After decades of extensive research, the *ZT* value for TE materials is still around 1 (ref. ^8^), prompting a need to explore alternative strategies to enhance STEG power generation. STEG outputs more power at higher Δ*T*, which can be achieved by increasing ${T}_{h}$ with efficient solar energy absorption and minimizing heat dissipation at the hot side, while lowering ${T}_{c}$ with efficient heat dissipation at the cold side^7,9^. STEGs could find potential applications in powering avionic devices^10^, wireless sensor networks^11^, wearable electronics^12^, and medical sensors^13^. However, these high-power-density (power/weight) applications require lightweight selective solar absorbers (SSAs) and heat dissipators for the STEG hot and cold sides, respectively.

An SSA needs to have high optical absorption in the solar spectral range (300–2500 nm) to harness as much solar energy as possible, but should have low emissivity in the IR (2.5–20 μm) to minimize radiation loss^14^. Extensive efforts have been made to optimize the optical performance of SSAs through material selection and structure design, such as utilizing photonic crystal absorbers^15,16^, multilayer film absorbers^17,18^, double ceramic layer absorbers^19,20^, and plasmonic absorbers^21,22^. Despite the notable advances in developing SSAs, their fabrication often requires high-vacuum thin-film deposition^17,18^ and photolithographic techniques^15,16^, which require multistep and time-consuming fabrication processes and complex equipment, thus restricting large-scale production at an affordable cost. Additionally, multi-layered absorbers have two inherent issues. First, SSA coatings usually have different thermal expansion coefficients from the substrate materials, which tends to compromise the SSA’s mechanical integrity at high temperatures^23^. Second, the typical multi-layered metal-dielectric coatings of SSAs have low thermal conductivity, which limits the heat transfer from the absorber to the STEG hot side. On the other hand, to increase STEG output power, complex and bulky cooling systems are often required to cool the cold side to increase Δ*T* ^4,7,24–26^. Among different cooling technologies^7,24^, solid-state passive cooling is the preferred one to save space and energy^25,26^. For passive cooling, large-sized metallic fins are generally used^25,26^ but they are bulky and limit STEGs in high-power-density applications. To reduce bulkiness, microscale fins have been developed using micromachining^27,28^ and photolithography^29^. However, microscale fins can only improve convective but not radiative heat dissipation^30,31^. For applications where radiation is the only path to dissipate heat, such as in space, these fin-based heat exchangers have very limited applications.

**Fig. 1:**
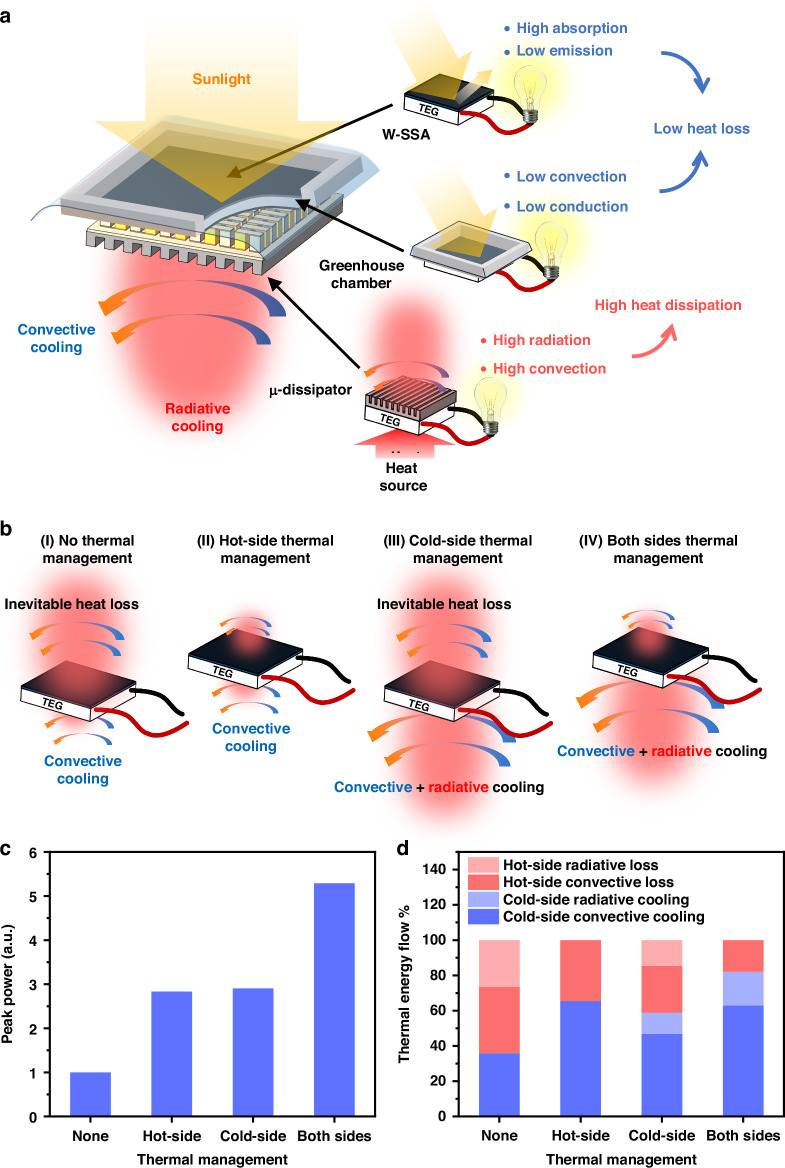
Theoretical design of spectral engineering and thermal management strategies for the STEG hot and cold sides **a** Schematic of enhancing STEG output power through hot- and cold-side thermal management. The hot-side thermal management system consists of a W-SSA and a greenhouse chamber to reduce heat loss. The cold-side thermal management system consists of a μ-dissipator, which enhances the cold-side heat dissipation. **b** Four cases of STEG with (I) no thermal management, (II) hot-side thermal management, (III) cold-side thermal management, and (IV) both sides thermal management. **c** Simulated STEGs’ peak output power with different thermal management strategies. **d** Simulated energy flows in the four STEGs. The blue bars represent the energy flow through the STEG

In this work, we develop a spectral engineering and thermal management strategy (Fig. 1a) that synergistically boosts the STEG power generation by 15 times with only a 25% increase in the STEG weight. At the hot side, we transform a regular tungsten (W) to a W-SSA through a femtosecond (fs)-laser processing technique. Additionally, we create a greenhouse chamber for W-SSA to minimize its convective heat loss. At the cold side, we use the fs laser processing to transform a regular aluminum (Al) to a super-high-capacity micro-structured heat dissipator (μ-dissipator) which improves the cold-side heat dissipation through both radiation and convection. In contrast to conventional multistep coating and micro-fabrication technologies, fs-laser processing is a single-step, scalable, and simple subtractive technique that can be applied to a range of materials of complex geometry, including metals^32,33^, semiconductors^34^, dielectrics^35^, glass^36^, and polymer^37^. Compared to other scalable nano- and micro-structuring techniques such as chemical etching and electrochemical deposition, fs laser processing is a pure physical approach, thus more environmentally friendly. The fs laser produced a range of nanostructures on W and microstructures on Al. By optimizing the size and density of these structures, at the hot side, we enhance solar absorption of W while minimizing its IR emissivity; at the cold side, we enhance IR emissivity across the entire blackbody radiation spectrum for Al and its surface area for heat dissipation. As a result, W-SSA has a significantly enhanced solar-to-thermal conversion efficiency, while Al μ-dissipator exhibits a superhigh heat-dissipation effect. We also numerically design and experimentally optimize the greenhouse chamber for W-SSA to minimize its convection and conduction heat losses to the ambient.

## Results

### STEG output power enhancement strategies

A STEG generates electrical power when there’s a Δ*T* across its hot and cold sides. The generated power is expressed as^38^

1 $${P}_{{STEG}}={\left({S}_{{TEG}}\varDelta T\right)}^{2}\frac{{R}_{L}}{{\left({R}_{L}+R\right)}^{2}}$$

where ${S}_{{TEG}}$ is the effective Seebeck coefficient of the TEG, *R* is the total electrical resistance between the TEG terminals, and ${R}_{L}$ is the load resistance. For a given TE material, an effective way to increase ${P}_{{STEG}}$ is to enlarge the Δ*T* across it. To achieve this goal, proper spectral and thermal managements at the hot and cold sides are needed.

To predict the effect of the hot- and cold-side thermal management, we conducted a numerical simulation of the heat transfer and thermoelectric effects for a STEG device (see Supplementary Note 1). Starting with a bare STEG attached with an ideal broadband solar absorber (BBA) and a regular metal heat dissipator, we investigated the enhancement of the STEG output power by reducing the inevitable thermal loss at the hot side and increasing the heat dissipation at the cold side (Fig. 1b). Assuming the hot-side thermal management minimizes the radiative loss and reduces the convective heat transfer coefficient by half. Similarly, we assume that the cold-side thermal management maximizes the radiative cooling and doubles the convective heat transfer coefficient. When the absorber converts solar energy to heat, part of the energy is lost due to hot-side radiation and convection. The rest is conducted through the STEG to the cold side, and this portion is utilized for power generation. Figure 1c shows the STEG peak output power for each case, and Fig. 1d shows the corresponding energy flow. The enhanced ${P}_{{STEG}}$ with thermal management is due to more thermal energy utilized by the STEG (represented by the blue bars). To have more thermal energy utilized by the STEG, the hot-side thermal loss needs to be minimized, while the cold-side heat dissipation needs to be maximized.

### Hot-side thermal management

As discussed before, the STEG output power is proportional to the Δ*T* across the TE material. The Δ*T* can be enhanced by maximizing the solar-thermal energy generation efficiency and minimizing the energy losses at the STEG hot side through effective spectral engineering and thermal management.

#### Spectral engineering

The manipulation of absorption/emission spectra of a solar absorber is essential for solar-thermal devices to minimize the radiative heat dissipation and maximize the solar-thermal energy conversion efficiency. An ideal solar absorber would exhibit near-complete absorbance within the solar spectrum while maintaining minimal thermal emittance in the IR region, characterizing it as an SSA^39^. In contrast, nonselective BBAs have inferior solar energy harvesting ability because of their significant radiative heat loss^40^. The creation of SSA that exhibits selective absorption over the solar spectrum (300–2500 nm) requires careful control of the hybridized surface plasmon resonances in the surface nanostructures. To determine the SSA material and the optimal fs-laser processing parameters, we create SSA on a variety of metals—nickel (Ni), copper (Cu), aluminum (Al), and W—using different laser power, scanning speed, and interline spacing. We measure their spectral absorption/emission (see Supplementary Note 2) using a UV-vis spectrometer and a Fourier transform infrared (FTIR) spectrometer (see details in the “Materials and methods” section), and we evaluate the solar absorption efficiency (${\eta }_{{abs}}$) as^40^

2 $${\eta }_{{abs}}=\bar{\alpha }-\frac{\bar{\varepsilon }\sigma \left({T}_{{abs}}^{4}-{T}_{{amb}}^{4}\right)}{C\bullet {I}_{{solar}}}$$

where $\bar{\alpha }$ and $\bar{\varepsilon }$ represent the average solar spectrum absorption and IR emissivity, respectively, $\sigma$ is the Stefan–Boltzmann constant, ${T}_{{abs}}$ and ${T}_{{amb}}$ are respectively the absorber temperature and ambient temperature, *C* and ${I}_{{solar}}$ represents the solar concentration and solar intensity.

**Fig. 2:**
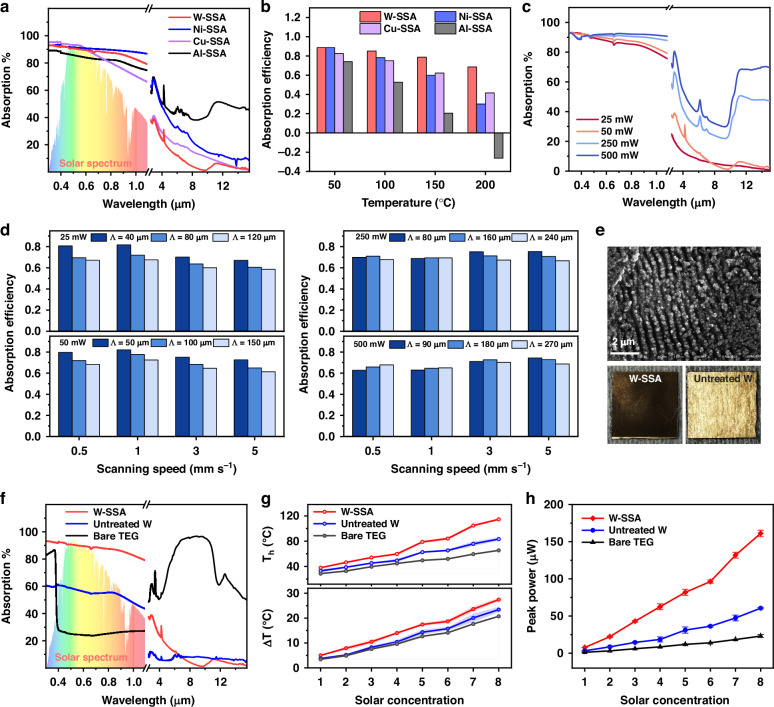
Spectral engineering for SSA fabrication and testing **a** Absorption spectra and **b** solar absorber efficiency ${\eta }_{{abs}}$ of optimized SSAs created on a variety of common metals: Ni, Cu, Al, and W. **c** Variation of absorption with different laser powers on W samples. The samples are densely patterned with a scanning speed of 1 mm s^−1^. **d** Solar absorber efficiency ${\eta }_{{abs}}$ for W-SSAs with different fs-laser processing parameters. **e** SEM and optical image of the optimized W-SSA. **f** Absorption spectra of three solar receivers: TEG’s ceramic absorber, untreated W, and W-SSA. **g** STEG’s hot-side temperatures (*T*_*h*_) and Δ*T* under 1–8× solar concentrations. **h** STEG peak power using the three solar absorbers 1–8× solar concentration

Figure 2a presents the spectral absorption/emission of the optimized W-SSA, Ni-SSA, Cu-SSA, and Al-SSA, and Fig. 2b shows the corresponding calculated ${\eta }_{{abs}}$ at different temperatures. A higher ${\eta }_{{abs}}$ suggests a higher solar energy absorption and lower thermal radiation loss, thus a more efficient SSA. Al is not a proper choice for SSA since a strong absorption peak at wavelengths >8 µm caused by the formed Al_2_O_3_ is inevitable^5^. Instead, Al is a good choice for a heat dissipator due to the strong emittance in the atmospheric transmission window. While Ni-SSA has high ${\eta }_{{abs}}$ at temperatures of 50 °C and 100 °C, its relatively high emission at shorter wavelengths leads to a quick drop of ${\eta }_{{abs}}$ as the temperature rises. On the other hand, Cu-SSA exhibits a relatively high solar absorption, a low IR emissivity, and a good ${\eta }_{{abs}}$ even at 200 °C. However, Cu has a relatively low melting point (~1000 °C), which could affect the stability of the fs-induced nanostructures as the nanoparticles have a much lower melting point than the bulk material^5^. We find that the optimized W-SSA exhibits a good spectral selectivity, and it is relatively stable ${\eta }_{{abs}}$ even at 200 °C due to the low spectral emission for wavelengths >6 µm. Therefore, we select W-SSA as the absorber because of its high melting point (3422 °C), high mechanical strength, and high flexibility of spectral manipulation.

The W-SSA’s high solar absorption and low IR emissivity originate from the fs-laser induced surface nanostructures. When light interacts with the electrons in these nanostructures, strong light absorption can occur through phenomena such as surface plasmonic resonance absorption^5^. Since the resonance frequencies depend on the size and density of nanostructures, and an ensemble of nanostructures broadens the convoluted plasmon resonances^5^, desired spectral absorption can be obtained by manipulating the size distribution of fs-laser induced surface nanostructures, which is done by altering the laser processing parameters. Figure 2c shows the variation of absorption with different laser powers. For densely laser-patterned W surfaces with the same scanning speed, we can make W-SSAs (25 mW and 50 mW samples) and W-BBAs (250 mW and 500 mW samples) by varying the laser power. Figure 2d shows the calculated ${\eta }_{{abs}}$ for W-SSA samples optimization. We find that the W-SSA sample that is fabricated using 50 mW laser power, 1 mm s^−^^1^ scanning speed, and 50 μm interline spacing has the highest ${\eta }_{{abs}}$. Figure 2e shows the SEM and optical images of the optimal W-SSA sample, and Fig. 2f shows the corresponding spectral absorption/emission of the W-SSA, untreated W, and the bare TEG. The optimized W-SSA exhibits a good solar spectrum weighted absorption of 0.9, which is about 3 times higher than a bare TEG with ceramic absorber (packaging material; $\bar{\alpha }=0.29$), maintaining an IR emissivity nearly as low as the untreated W ($\bar{\varepsilon }=0.11$).

To test the effectiveness of the optimized W-SSA, we attach the W-SSA or an untreated W solar absorber to the STEG hot side and measure the hot-side temperature and corresponding output power. The bare TEG with a ceramic package at the hot and cold sides is used as a control. The solar absorber and thus the STEG hot side is heated using a concentrated light beam from a calibrated solar simulator, while the STEG cold side is cooled by natural convection. Figure 2g shows the corresponding hot-side temperatures and Δ*T*. Due to the increase in absorbed solar energy and reduced radiation loss, the STEG hot-side temperature is raised and Δ*T* is enlarged. As a result, the W-SSA integration shows ~7× and ~2.7× enhancements in STEG peak power, at all solar concentrations, compared to the bare TEG and regular W absorber, respectively, as shown in Fig. 2h.

#### Convective-conductive thermal management

**Fig. 3:**
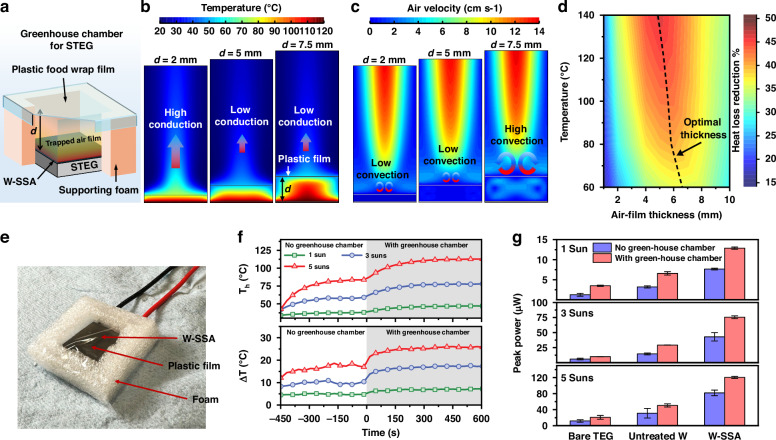
Numerical design and experimental realization of a greenhouse chamber for the STEG hot-side thermal management **a** Schematic of the greenhouse chamber for hot-side convective thermal management. Simulated **b** temperature distributions and **c** air velocity fields for air-film thicknesses *d* = 2 mm, 5 mm, and 7.5 mm. **d** Optimization of the air-film thickness to minimize heat loss through simulations. **e** Photo of STEG with a 6-mm thick greenhouse chamber. **f** Hot-side temperature (*T*_*h*_) and Δ*T* for the W-SSA-integrated STEG with and without the greenhouse chamber under 1–5× solar concentrations. **g** STEG peak output power with and without the greenhouse chamber under 1–5× solar concentrations

While the utilization of W-SSA maximizes the solar absorption efficiency and minimizes the spectral thermal loss, the convective loss from the SSA still needs to be minimized. In natural convection, air in contact with a hot surface gets heated and rises above the surface. Relatively cold surrounding air fills the gap, and this air circulation process dissipates heat away from the surface. A vacuum environment is commonly used to reduce the thermal convection^1,4^. However, it involves a complicated system setup and heavy and costly components. Aerogel is an alternative way to reduce thermal convection^41^, but its fabrication is complicated and time-consuming. Convection loss from a heated surface can be minimized by minimizing the air circulation between the surface and the ambient. As air has a thermal conductivity as low as 0.03 W m^−^^1^ K^−^^1^ (at 100 °C), it can serve as an excellent insulation material. Inspired by bubble sheet that uses air pockets to reduce convection losses, we numerically design and experimentally realize a greenhouse chamber for the W-SSA by trapping an air film, as illustrated in Fig. 3a. A thinner air film can prevent air circulation effectively and reduce the convection loss, but its thin thickness would cause thermal loss through conduction. Therefore, the air-film thickness is crucial to achieve good thermal insulation. We numerically optimize the greenhouse chamber thickness $d$ using a computational fluid dynamics simulation (see Supplementary Note 3). As shown in Fig. 3b, c, a 2-mm-thick air film can significantly reduce air circulation, reducing the convective heat transfer. However, its thin thickness cannot effectively stop the conductive heat transfer, resulting in a small temperature gradient across the air film. In contrast, a 7.5-mm-thick air film has enough thickness to stop conductive heat transfer, but its large space allows more air circulation and deteriorates the insulation effect. For a 5-mm-thick air film, heat transfer through conduction and convection can both be suppressed, thus maximizing the insulation effect. Figure 3d shows the effect of air-film thickness on suppressing the convective/conductive heat loss from a hot surface at different temperatures. For a STEG with a hot-side temperature of about 95 °C, a 6-mm-thick air film can provide >40% reduction in the convective/conductive thermal loss. The experimental result also verifies the existence of such optimal air-film thickness and its thermal insulation effect (Fig. S3d).

For experimental demonstration, we create such a greenhouse chamber using a stretch-tight plastic food wrap film made of polyethylene, as shown in Fig. 3e. Like the bubble sheets, the air trapped by the plastic film served as a good insulation to reduce the heat loss. Instead of using bi-layered bubble sheets, we raise a single layer of plastic film from the STEG hot side using a thermally insulating spacer. This design has two advantages: the single-layer design minimizes the transmission loss of solar radiation, and the elevated plastic film is away from the heated surface, avoiding potential damage to the plastic film and enriching the film’s material choice. Figure 3f shows the Δ*T* and hot-side temperature for the W-SSA integrated STEG with and without a 6-mm thick greenhouse chamber. Enhancement in the Δ*T* increases with solar concentration and shows a ~45% increase at 5-sun solar concentration. Figure 3g shows the STEG peak output power with and without the greenhouse chamber at different solar concentrations. For lower concentrations (1 sun and 3 suns), the greenhouse chamber increases the peak power by ~1.7×, resulting in a ~10× enhancement in the STEG output power compared to the case without any hot-side thermal management. At a 5-sun solar concentration, the effect of greenhouse chamber on the STEG power enhancement is lower as compared to 1- and 3-sun solar concentrations (Fig. 3g) due to an increase in the working temperature higher than the optimal temperature for the TEG material (Bi_2_Te_3_ in this case).

### Cold-side spectral engineering and thermal management

While the hot-side thermal management has been demonstrated to be capable of significantly enhancing the STEG's power output, the heat-dissipation capability at the cold side is also important for increasing the STEG’s output power. As the STEG efficiency ${\eta }_{{STEG}}$ is proportional to $\varDelta T/{T}_{h}$, an efficient cold-side thermal management can not only enlarge the Δ*T*, but also lower the ${T}_{h}$ of the STEG device, further improving its solar-to-electrical conversion efficiency^7^. Typically, a bulky heat sink is attached at the cold side of a TEG/STEG to dissipate heat through convection (${Q}_{c}$). Although the bulky heat sink can have high convective heat dissipation, its large size may not be suitable for devices with size and weight restrictions. Our STEG cold-side thermal management system involves a μ-dissipator that dissipates heat through convection and radiation. The μ-dissipator is directly fabricated on a thin Al foil using fs-laser surface nano-/micro-structuring. Compared with a regular Al surface (Al dissipator), the μ-dissipator surface has hierarchical structures where surfaces of the fs-laser written micro-grooves and micro-ridges are covered with nano-/micro-scaled structures. Patterns and dimensions of the micro-grooves and micro-ridges (surface geometry) determine the heat transfer coefficient and solid-air interface area for the convective heat dissipation, while the size and density of nano-/micro-structure (surface morphology) control IR emissivity and thus the radiative heat dissipation (${Q}_{R}$). Therefore, the optimization of the μ-dissipator aims to maximize the convection and radiation combined overall heat-dissipation capacity.

**Fig. 4:**
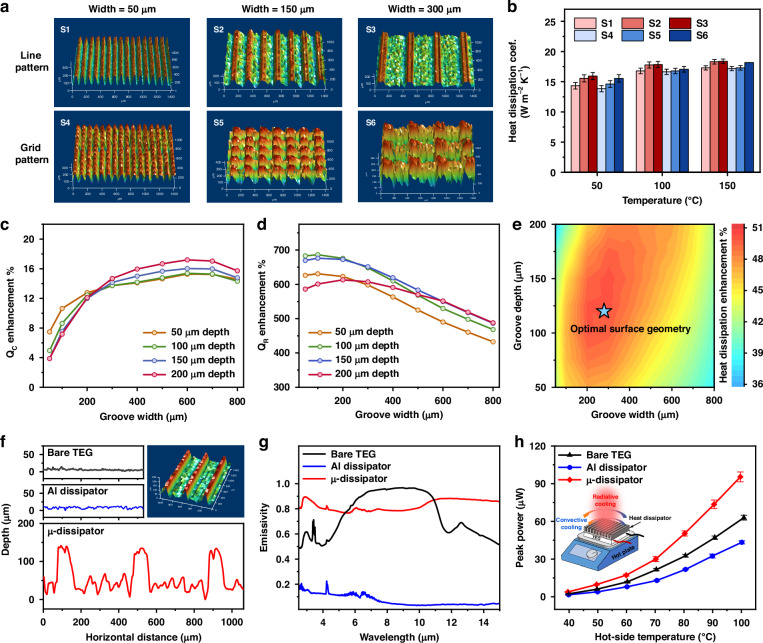
Micro-heat-dissipator design, fabrication, and testing for the STEG cold-side thermal management **a** 3D surface profiles of the μ-dissipators with different surface geometries. Two types of patterns are created: the line pattern (S1, S2, and S3) and the grid pattern (S4, S5, and S6). For each pattern, three different groove widths of 50 μm, 150 μm, and 300 μm are fabricated, and the groove depth is maintained at ~100 μm. **b** Heat-dissipation coefficient for μ-dissipators with different surface geometries measured under different temperatures. Simulated **c** convective cooling power (${Q}_{c}$), **d** radiative cooling power (${Q}_{R}$), and **e** overall cooling capacity enhancements for μ-dissipator with different surface topographies at 100 °C. **f** Surface profiles of the bare TEG, Al dissipator, and μ-dissipator. The insert is the 3D view of the μ-dissipator. **g** Absorption spectra of the TEG’s ceramic dissipator, Al dissipator, and μ-dissipator. **h** STEG peak output power with different cold-side heat dissipators when the hot-side temperature is at 40–100 °C

To investigate the effect of surface geometries on μ-dissipator’s cooling performance, we compare two fs-laser scanning patterns: the line pattern and the grid pattern (Fig. 4a). The line pattern is obtained by line-by-line laser scanning, and the grid pattern is obtained by performing a line-by-line scan in the horizontal direction, followed by the same line-by-line scan in the perpendicular direction. For each pattern, samples with groove widths of 50 μm, 150 μm, and 300 μm are fabricated, and the groove depth is maintained at ~100 μm. We use a Joule heater and a DC power source to measure the test sample’s cooling performance at different temperatures (see Supplementary Note 4). Figure 4b illustrates the heat-dissipation coefficient for each sample, which rises with temperature as radiative cooling becomes increasingly significant at higher temperatures. Overall, samples with line patterns (S1, S2, and S3) have slightly better cooling performance than the grid patterns (S4, S5, and S6). It’s probably because hot air cannot efficiently escape from the dips formed at the intersections of the laser scanning paths on grid patterns. Additionally, the μ-dissipator’s cooling performance improves as the groove width increases from 50 μm to 300 μm. A significant rise in cooling performance is observed between groove widths of 50–150 μm, while the increase from 150 μm to 300 μm is relatively smaller. Therefore, since the line pattern has a higher cooling power than the grid pattern, and its fabrication time is only half of the grid pattern, we choose the line pattern for the μ-dissipator fabrication and further surface geometry optimization.

To optimize the μ-dissipator’s surface geometry, we perform a numerical simulation to compute enhancement in the convective and radiative cooling power of the μ-dissipator, approximating the surface geometry as rectangular micro-grooves, as compared to a regular Al dissipator. The radiative cooling power is computed using experimental-data-approximated IR emissivity (see Supplementary Note 5). Figure 4c shows the effect of μ-dissipator surface geometry (depth and width) on the convective cooling power enhancement over a regular Al foil at 100 °C. A μ-dissipator with deeper and narrower micro-grooves has a larger surface area. However, air flow inside the deep and narrow grooves may be stagnated due to air viscosity, causing a lower heat transfer coefficient of convective heat dissipation^30,31^. As indicated by simulation, micro-grooves with a width between 400 and 800 μm and with a depth larger than 100 μm achieve a better balance between enhancing convective air flow and increasing heat-dissipation surface area. Figure 4d shows the corresponding enhancement in the radiative cooling power at 100 °C. Different surface topographies require different fs-laser parameters to fabricate, which alters the surface morphology and hence affects the IR emissivity. Also, the multiple reflection of light beams inside narrow grooves enhances the light absorption and thus the IR emissivity, while such multiple reflection is reduced in very wide grooves, leading to lower IR emissivity. As a result, despite a slight decrease in IR emissivity for grooves deeper than 150 μm, densely packed narrower grooves tend to have stronger radiative cooling power. Combining the convective and radiative cooling effects, as shown in Fig. 4e, a micro-groove with a width from 200 to 300 μm and a depth from 100 to 150 μm is ideal to achieve the optimal overall heat-dissipation capacity.

We fabricate a 20 mm × 20 mm Al μ-dissipator with the optimized surface geometry (Fig. 4f). The fabricated μ-dissipator has a groove depth of 120 μm and a groove width of 320 μm, resulting in an effective surface area increment of ~180% compared to a regular Al dissipator. In addition, the micro- and nano-scale surface structures created by fs-laser processing enhances light absorption due to surface plasmon resonance across a broad spectral range, leading to an enhancement in IR emissivity of ~7.2× compared to a regular Al dissipator, as shown in Fig. 4g. Overall, the μ-dissipator exhibiting ~2× enhancement in cooling performance compared to a regular Al dissipator (Fig. S5e).

We test the heat-dissipation performance of the μ-dissipator for a compact STEG device by comparing the electrical output power of the three STEGs at various ${T}_{h}$: a bare TEG, a TEG integrated with a regular Al dissipator on the cold side, and a TEG integrated with the optimized μ-dissipator on the cold side. The TEGs are placed on a hotplate with adjustable temperature, and the Al dissipator and μ-dissipator have the same dimensions as the TEG. As shown in Fig. 4h, in the entire TEG operating temperature range (40–100 °C), the μ-dissipator integrated STEG generates ~2.3× higher peak power than the STEG integrated with a regular Al dissipator. The bare TEG produces a slightly higher output power compared to the Al dissipator integrated TEG because the ceramic dissipator (packaging material) of the TEG has stronger radiative thermal emission than the regular Al surface. At low temperatures, the role of radiative cooling decreases, allowing convective cooling to become the primary heat-dissipating mechanism. The μ-dissipator surpasses both the Al-dissipator-integrated STEG and the bare STEG due to its larger surface area, which enhances convective cooling at lower temperatures despite minimal radiative cooling. As the temperature rises, both radiative and convective cooling contribute to heat dissipation. The μ-dissipator’s enhanced convective and radiative cooling makes it suitable for both low and high-temperature heat-dissipation applications.

### Synergistic effect of STEG hot- and cold-side spectral and thermal management

**Fig. 5:**
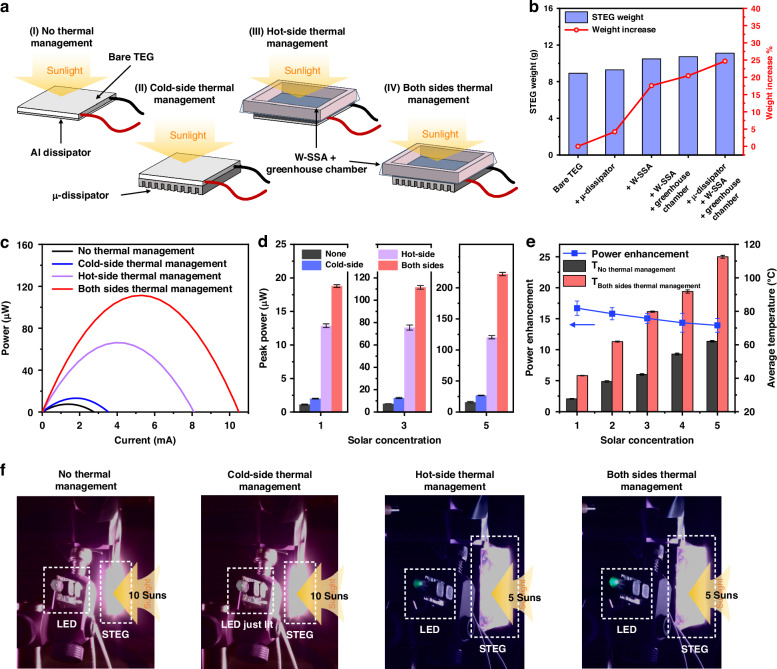
Synergistic effect of STEG hot- and cold-side spectral and thermal management **a** Schematics of four cases of STEG with different thermal management strategies. **b** STEG weight increases when adding the μ-dissipator, W-SSA, and greenhouse chamber to the TEG. **c** STEG power-current curves under 3 suns. **d** STEG peak output power under 1–5× solar concentrations. **e** STEG power enhancement and TEG average temperatures under 1–5× solar concentrations by applying spectral and thermal management on both sides. **f** Photos of LED illumination when powered by the four STEGs in (**a**)

After independently designing and optimizing the STEG hot- and cold-side thermal management systems and testing their performance, we assembled the final STEG by integrating the optimized W-SSA surface on the hot side and the optimized μ-dissipator on the cold side. To better investigate this synergistic enhancement, we choose four STEG devices for comparison: a bare STEG integrated with regular Al dissipator as control (case of no thermal management), a STEG with only cold-side thermal management, a STEG with only hot-side thermal management, and a STEG with both hot- and cold-side thermal management (Fig. 5a). For the STEG with thermal management on both sides, the weight of the system only increases 25% due to the low weight of the components contained in the device, as shown in Fig. 5b. Figure 5c shows the typical power-current curves of the four cases under 3 suns, and Fig. 5d shows the STEG output peak power at different solar concentrations. A peak output power enhancement of ~1.3× is observed for the STEG with cold-side thermal management, and ~10× enhancement is observed for the STEG with hot-side thermal management. When applying both the hot- and cold-side thermal management, a peak output power enhancement of ~15× is observed.

Figure 5e presents the ratio of STEG power enhancement by applying spectral and thermal management on both sides. The power enhancement ratio decreases slightly at higher solar concentrations (from ~17× at 1 sun to ~14× at 5 suns), even though the system efficiency of the case with spectral and thermal management increases with solar concentrations (Fig. S8a). It is probably due to a decrease in TE material efficiency as temperature rises. The thermoelectric efficiency of a typical Bi_2_Te_3_-based TEG peaks near room temperature and decreases as temperature rises^42^. As solar concentration increases, the average STEG temperature for the case with both-side thermal management rises more quickly than its counterpart, leading to a lower power enhancement ratio, even though the spectral and thermal management system performs better at higher temperatures. Thus, the drop in STEG power enhancement can be resolved by utilizing TE materials with a higher working temperature.

STEG technology has potential applications in powering wireless sensors for the Internet of Things (IoT), powering wearable devices, and serving as off-grid renewable energy systems in rural areas. For instance, the IoT connects numerous sensor nodes in the physical world, allowing real-time data collection and immediate response to events in fields like weather, healthcare, and transportation^43^. Combining with STEG technology, the sensor nodes can be self-powered, eliminating the need for batteries or a power grid. To illustrate the STEG power enhancement and demonstrate the capability of providing sufficient power using our spectral engineering and thermal management strategy, we apply the four STEGs in Fig. 5a to power LEDs, as shown in Fig. 5f. Without thermal management, the STEG cannot light the LED even under 10 suns. With cold-side thermal management, the STEG provides power to merely light the LED. With the hot-side thermal management, the STEG provides enough power to light the LED at only half of the solar concentration (5 suns). The combination of hot- and cold-side thermal management enables the STEG to power the LED at its maximum brightness at 5-sun solar concentration.

## Discussion

In this study, we present a strategy to enhance the STEG output power by applying spectral and convective thermal management on both hot and cold sides. Through spectral engineering, the fs laser processed W-SSA exhibits high solar spectral absorption and low IR emission, substantially decreasing the radiative energy loss. Along with the hot-side greenhouse chamber enclosed by plastic films, both the radiative and convective thermal losses from the hot side are effectively suppressed, and the STEG output power is boosted by ~10× compared to a bare TEG without thermal management. Through fs laser surface morphology modification, the μ-dissipator exhibits ~2× cooling performance over a regular Al dissipator and leads to 2.3× STEG output power enhancement compared to the Al dissipator under the same temperature. When applying both the hot- and cold-side thermal management, with the minimized hot-side radiative and convective heat losses and the enhanced cold side radiative and convective cooling capacity, more solar energy is conducted through and utilized by the STEG. This results in a greater Δ*T* across the STEG and hence an output power increase of over 15× while maintaining device compactness with only a 25% increase in weight. Table 1 compares this work with previous works in STEG power enhancement^5,33,41,44–49^, and our spectral engineering and thermal management strategy shows superior advancement in power enhancement while maintaining the compactness and lightweight of the system.

**Table 1:** Comparisons of this work with previously reported ones

Spectral and thermal management strategy	STEG power enhancement	Comment	Ref.
CNT solar absorber on the hot side	~3.5× compared to bare TEG	/	^44^
Electrochemically deposited Nickel-tin SSA on the hot side	~4.5× compared to the untreated Cu absorber	Water cooling on the cold side	^45^
Fs-laser-treated Al solar absorber on the hot side	4 to 9× compared to bare TEG and untreated Al absorber	Regular heat sink on the cold side	^46^
Fs-laser-treated W-SSA on the hot side	~2.3× compared to the untreated W absorber	Regular heat sink on the cold side	^5^
Transparent aerogel window on the hot side	1.17× compared to uncovered STEG	1.71× power enhancement under 3 m s^−^^1^ wind	^41^
TEG with SSA attached to dye-sensitized solar cell (DSSC)	~1.5× compared to DSSC along	Bulky heat sink and ice water cooling on the cold side	^47^
PMMA-based radiative heat dissipator on the cold side	~6× compared to bare TEG	Multilayer solar absorber on the hot side	^48^
Fs-laser-treated Al heat dissipator on the cold side	2.8× compared to the untreated Al heat dissipator	/	^33^
Anodic aluminum oxide heat dissipator on the cold side	2× compared to the untreated Al heat dissipator	1.55–1.7× compared to a commercial heat sink	^49^
Fs-laser-treated W-SSA + greenhouse chamber + μ-dissipator	~15× compared to bare TEG with Al heat dissipator	/	This work

With the enhanced power generation, a small STEG can potentially power microelectronic devices, such as autonomous sensors for weather monitoring and agriculture applications^11^, and smart devices, which typically run at μW to mW power levels^13^. Moreover, STEGs can be combined with spectral splitting hybrid PV-TE systems^50^ and dye-sensitized solar cells^47^ to further enhance the output power for more energy-demanding devices. Our work demonstrates the significant potential of direct fs-laser processing in enhancing STEG performance and opens the possibility of applying STEG technology to various applications, such as powering IoT sensors and wearable devices, and it shows the potential to be combined with PV cells to further enhance the solar energy harvesting efficiency.

## Materials and methods

### Direct fs-laser surface processing

The W-SSA and μ-dissipator are created on the W and Al surface with direct fs-laser processing. W sheets are purchased from McMaster with a thickness of 250 μm and a purity of 99.99%. Polished Al foils are purchased from Goodfellow with a thickness of 200 μm and a purity of 99.99%. Samples are cut into squares with a size of 20 mm × 20 mm to match the size of the TEG module, degreased and washed with acetone and deionized water in an ultrasonic cleaner, respectively, before the laser processing. The laser used in our experiments is a Ti: sapphire fs-laser, which delivers horizontally polarized pulse trains at a repetition rate of 1 kHz with a central wavelength of 800 nm and a pulse duration of 60 fs. The maximum average power delivered by the laser system is 7 W. The pulse energy is controlled using a combination of a half-wave plate and a polarizer. The laser is focused by a lens with a focal length of 200 mm and incident at normal incidence. The samples are mounted at a computerized XYZ precision stage and processed by raster scanning the laser beam at different speeds across the sample. To explore the fs-laser fabrication window of W-SSA, laser power is varied from 25 to 500 mW, scanning speed is varied from 0.5 to 5 mm s^−^^1^, and interline period is varied from 40 to 270 μm. To create the μ-dissipator that matches the optimized geometry of the surface morphology, a laser power of 3 W, a scanning speed of 1 mm s^−^^1^ are used.

### TEG output measurements

Low-cost commercial Bi–Te based TEG module bought from Amazon, with a size of 20 mm × 20 mm, is used in the experiment. For different STEG configurations, W-SSA, untreated W, μ-dissipator, and Al dissipator are integrated to the TEG hot and cold sides by a high-temperature thermal conductive paste (OMEGATHERM 201). To measure the power enhancement of STEG integrated with W-SSA and μ-dissipator, an indoor experiment is performed using a solar simulator with an AM 1.5 airmass filter. A plano-convex lens with a 250 mm focal length and 150 mm diameter is mounted at the output port of the solar simulator to enhance the solar concentration. The solar simulator power is adjusted to achieve solar irradiances ranging from 1 to 10 suns (1–10 kW m^−^^2^). To measure the heat-dissipation capability of μ-dissipator integrated TEG, an indoor experiment is performed using a hotplate by setting the temperature ranging from 40 to 100 °C, covering the STEG temperatures under various solar concentrations in this study. The μ-dissipator and Al dissipator are integrated into the TEG cold side, and the hot side is in contact with the hotplate. TEG output measurements are taken after the sample is connected and the TEG temperature becomes stable for at least 10 min. TEG power is measured using a Keithley-2400 source meter by sweeping the voltage from the open-circuit voltage to 0 while measuring the current. Each measurement is repeated four times. The TEG hot- and cold-side temperature is monitored using thermocouples (TP-01 Type K) and a data logger (Omega TC-08). The mass of STEG with simultaneous integration of W-SSA and μ-dissipator is measured using a balance (Radwag) with an accuracy of 0.001 g.

### Surface morphology and optical properties characterization

To characterize the spectral absorptance/emittance of W-SSA and μ-dissipator in the UV-vis-NIR region, we measure the total hemispherical optical reflection of the samples using an ultraviolet-visible (UV-vis-NIR) PerkinElmer Lambda 900 spectrophotometer. For the spectral absorptance/emittance in the IR region, we use Fourier transform infrared (FTIR) spectroscopy, a Bruker IFS 66/S FTIR spectrometer, each equipped with an integrating sphere. The measurement is performed at room temperature, and the absorption spectra are assumed to be constant at higher temperatures. The transmission of the plastic film is characterized using the same spectrometers without the integrating spheres to take solar power loss due to scattering into account. The surface morphology of W-SSA and μ-dissipator is analyzed by scanning electron microscopy (SEM) and three-dimensional (3D) laser scanning microscope (Keyence VK-X700). The SEM is a Zeiss–Auriga field emission operating at an accelerating voltage of 20 kV.

## Supplementary information

Supplementary Information for 15-fold Increase in Solar Thermoelectric Generator Performance Through Femtosecond-laser Spectral Engineering and Thermal Management

## Data Availability

The data that supports the findings of this study are available from the corresponding author upon reasonable request.
