# Supplementary material for: 15-Fold increase in solar thermoelectric generator performance through femtosecond-laser spectral engineering and thermal management
Source: Light Sci Appl. 2025 Aug 12;14:268. doi: 10.1038/s41377-025-01916-9 (PMC12340015; doi:10.1038/s41377-025-01916-9)
Supplement: Supplementary file 1 — Supplementary Information for 15-fold Increase in Solar Thermoelectric Generator Performance Through Femtosecond-laser Spectral Engineering and Thermal Management [file 41377_2025_1916_MOESM1_ESM.docx]

**Supplementary Information for**

**15-fold Increase in Solar Thermoelectric Generator Performance Through Femtosecond-laser Spectral Engineering and Thermal Management**

Tianshu Xu, Ran Wei, Subhash C. Singh*, Chunlei Guo*

The Institute of Optics, University of Rochester, Rochester, NY-14627, USA

*Corresponding author: [ssingh49@ur.rochester.edu](mailto:ssingh49@ur.rochester.edu), [guo@optics.rochester.edu](mailto:guo@optics.rochester.edu)

**Supplementary Note 1: STEG heat transfer and thermoelectric effect simulation**

A solar thermoelectric generator (STEG) is a device that converts solar thermal energy directly into electrical energy. When a temperature gradient is applied across the junctions of two different materials, typically n-type and p-type, an electric current is produced. A schematic of this effect for a TE unit is illustrated in Figure S1a. Taking the heat conduction, Joule heating and thermoelectric effects into account, the heat fluxes through the STEG hot side ($Q_{h}$) and cold side ($Q_{c}$) are expressed as ^1, 2^

| $Q_{h}=S T_{h} I-\frac{1}{2} I^{2} R+K(T_{h}-T_{c})$ | (S1.1) |
| --- | --- |
| $Q_{c}=S T_{c} I-\frac{1}{2} I^{2} R+K(T_{h}-T_{c})$ | (S1.2) |

where $S$ is the Seebeck coefficient, $T_{h}$ and $T_{c}$ are the hot- and cold-side temperatures, $R$ is the electrical resistance, $I$ is the electrical current, and $K$ is the thermal conductance. According to the first law of thermodynamics, the TE unit generates an electric power $P_{TE}$ equals to the difference between the hot- and cold-side heat fluxes, and thus relates to the temperature gradient $\Delta T=T_{h}-T_{c}$ across the TEG as

| $P_{TE}=Q_{h}-Q_{c}=S I \Delta T-I^{2} R$ | (S1.3) |
| --- | --- |

A numerical model for a TEG module is developed using finite element analysis in COMSOL, which involves governing equations of heat transfer in solids, electric currents, and thermoelectric effects. The heat transfer in solid interface is used to model heat transfer in solids by conduction, convection and radiation using a governing equation of

| $\rho C_{p}\boldsymbol{u}\cdot\nabla T+\nabla\cdot\boldsymbol{q}=Q_{ted}+Q$  $\boldsymbol{q}= -K\nabla T$ | (S1.4) |
| --- | --- |

where $\rho$ is the density, $C_{p}$ is the specific heat, $\boldsymbol{u}$ is the translation motion velocity, $\boldsymbol{q}$ is the conduction heat flux, $Q_{ted}$ is the thermoelastic effect, and $Q$ is the heat source. The physics interface of electric currents solves the Ohm’s law based current conservation equations

| $\nabla J=Q_{j}$ $J=\sigma E+J_{e}$ $E=-\nabla V$ | (S1.5) |
| --- | --- |

where $J$ is the induced electric current, $Q_{j}$ is the current source, $E$ is the electric field, $\sigma$ is the electric conductivity, $J_{e}$ is the external current source, and $V$ is the electric potential. The physics interface of thermoelectric effects combines the heat transfer in solids and electric current interfaces to model Peltier-Seebeck-Thomson effects that can be described as

| $q=P J$ $P=S T$ $J_{e}=-\sigma S \nabla T$ | (S1.6) |
| --- | --- |

where $P$ is the Peltier coefficient, and $S$ is the Seebeck coefficient.

The numerical model simulates STEG with a module dimension of 20 mm × 20 mm × 4 mm, which consists of 45 thermocouple TE units, as shown in Figure S1b. The TE units are made of P-type and N-type bismuth telluride Bi_2_Te_3_ with a leg dimension of 1 mm × 1.2 mm × 2.3 mm. The TE units are connected electrically in series and thermally in parallel. The top planar surface is defined as the STEG hot side with an inward heat flux representing the absorbed solar energy, and a convective loss and a radiative loss are also defined on this surface. The bottom planar surface is defined as the STEG cold side with both convective and radiative cooling defined on this surface. One end of the STEG leg is defined as terminal and the other end is defined as the ground. We considered the heat to flow from the hot side and dissipated from the cold side without thermal loss from the STEG side walls. A stationery study is implemented to the STEG model to predict the electrical power generation and the heat flows with different thermal management strategies. Figure S1c,d show typical power-current and voltage-current curves of the STEG.

The strategies of enlarging $\Delta T$ can be considered from the power flow perspective. The solar energy absorbed by STEG dissipates to the surroundings through convection, radiation, conduction, and electric power generation. For an ideal case that neglects the conduction loss, the power balance of the STEG can be approximated as

| $P_{solar}=P_{conv}+P_{rad}+P_{cond}+P_{TEG}\approx P_{conv, hotend}+P_{rad, hotend}+P_{conv, coldend}+P_{rad, coldend}+P_{TEG}$ | (S1.7) |
| --- | --- |

where the $P_{solar}$ represent absorbed solar power, $P_{conv}$ is the convective heat dissipation, $P_{rad}$ is the radiative heat dissipation, $P_{cond}$ is the conductive heat dissipation, $P_{TEG}$ is the electric power generated by TEG. These powers are expressed as

| $P_{solar}=\alpha\cdot C\cdot I_{solar}$ | | (S1.8) |
| --- | --- | --- |
| $P_{conv, hotend}=h_{hot-end} A (T_{h}-T_{amb})$ | $P_{conv, coldend}=h_{coldend} A (T_{c}-T_{amb})$ |  |
| $P_{rad, hotend}=\varepsilon\sigma A (T_{h}^{4}-T_{amb}^{4})$ | $P_{rad, coldend}=\varepsilon\sigma A (T_{c}^{4}-T_{amb}^{4})$ |  |

where $\alpha$ represent the solar absorptance, $C$ represents the solar concentration, and $I_{solar}$ represents the solar intensity, $h$ denotes the convective heat transfer coefficient, $\varepsilon$ denotes the IR emissivity, $A$ represents the cross-section area, $\sigma$ is the Stefan-Boltzmann constant, $T_{amb}$ is the ambient temperature. According to the law of heat conduction, $\Delta T$ is related to the heat flow through the STEG $Q_{TEG}$ as

|  | $\Delta T=Q_{TEG}\cdot\frac{d}{K\cdot A}$ | (S1.9) |
| --- | --- | --- |

where $d$ is the thickness of STEG, and $K$ is the TEG’s average thermal conductivity. As $Q_{TEG}$ is the net power flow through the STEG, it is approximately equal to the heat flow dissipated from the cold side as other energies are lost from the hot side, assuming no heat loss from the STEG side walls and a negligible electrical power output.

| $Q_{TEG}\approx P_{conv, coldend}+P_{rad, coldend}=P_{solar}-P_{conv, hotend}-P_{rad, hotend}$ | (S1.10) |
| --- | --- |

Thus, maximizing the heat flow through the STEG is the key to maximizing its output power, which requires thermal management to maximize the solar power absorption and minimize the convective and radiative loss from the hot side. Meanwhile, enhancing the cold side convective and radiative cooling capacity can also lead to more heat flow through STEG, boosting the output power.

To better understand the roles and contributions of hot- and cold-side thermal management, this model is also used to conduct simulations of the STEG with different thermal management strategies to monitor the energy flows, as shown in Figure S1e. For the case with no thermal management, most of the energy (70%) is wasted through sun light reflection from the white ceramic surface, and only 12% is conducted through and utilized by STEG. When applying cold-side thermal management, the solar energy utilized by the STEG increases to 15%. When applying hot-side thermal management, the hot-side energy loss is greatly reduced, resulting in 52% of the solar energy utilized by STEG. Finally, with thermal management applied to both sides, over 60% of the energy is utilized by STEG, leading to the great enhancement in the STEG output power.


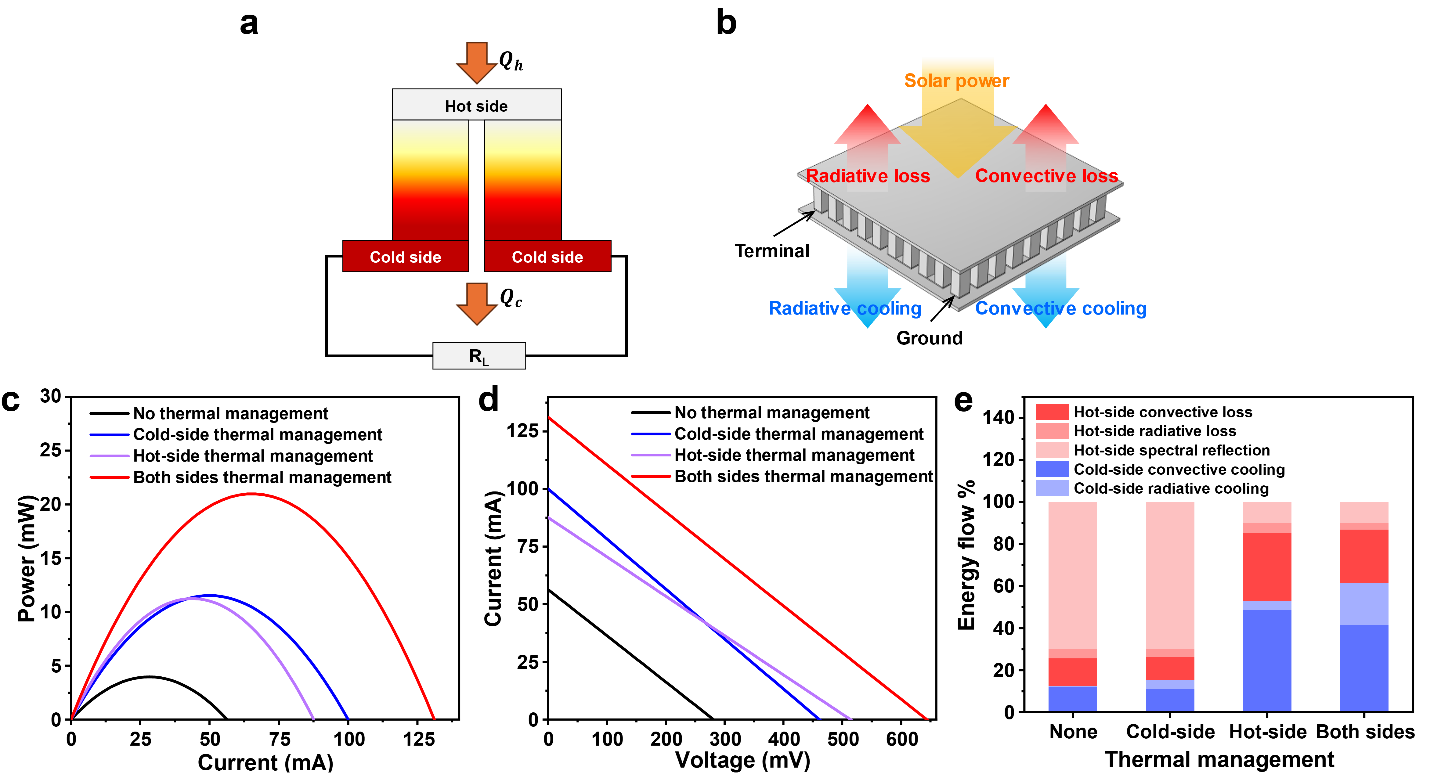


**Figure S1:** (a) Schematic of thermoelectric effect in a TE unit. (b) STEG model for numerical simulation. Typical simulated (c) power-current and (d) voltage-current curves for different thermal management strategies. (e) Simulated energy flow for the four cases of STEG with different thermal management strategies.

**Supplementary Note 2: Spectral optimization of selective solar absorber (SSA)**

To find the optimum fs-laser processing parameters to create SSAs, we vary the laser power, scanning speed and the interline period to create different surface structures on nickel (Ni), copper (Cu), aluminum (Al), and tungsten (W) foils. We analyze the solar spectrum averaged absorption and IR emissivity of different fabricated samples. The solar spectrum averaged absorption of the light absorber is determined as follows:

|  | $\bar{\alpha}=\frac{1}{I}\int_{0}^{\infty} d\lambda\varepsilon\left( \lambda\right)\frac{dI}{d\lambda}$ | (S2.1) |
| --- | --- | --- |

And the IR emissivity ($\bar{\varepsilon}$) is calculated as

|  | $\bar{\varepsilon}=\frac{\int_{0}^{\infty} {d\lambda\varepsilon\left( \lambda\right)}/\left\{ \lambda^{5}\left[ exp\left( {hc}/{\lambda kT} \right)-1 \right] \right\}}{\int_{0}^{\infty} {d\lambda}/\left\{ \lambda^{5}\left[ exp\left( {hc}/{\lambda kT} \right)-1 \right] \right\}}$ | (S2.2) |
| --- | --- | --- |

where $I$ is the solar intensity, $\lambda$ is the wavelength, $\varepsilon\left( \lambda\right)$ is the spectral absorptance of the light absorber, $\frac{dI}{d\lambda}$ is the spectral light intensity of the ai-mass coefficient (AM) 1.5 solar spectrum, $h$ is Plank’s constant, $c$ is the speed of light, $k$ is the Boltzmann constant, and $T$ is the absorber temperature, which is taken as 100 ℃. Figure S2a,b shows the calculated solar absorptance and emissivity for W-SSA samples with different laser processing parameters. Representative data of the variation in spectral absorptions with laser scanning speed for W-SSA, Cu-SSA, Ni-SSA, and Al-SSA is shown in Figure S2c-f.

For W-SSA, the absorption bandwidth of the surface becomes wider as a higher laser power and lower scanning speed, suggesting the formation of nanostructures with lager size and higher density ^3^. To obtain an absorption band that matches the solar spectrum, lower laser power and higher scanning speed needs to be used to create smaller nanostructures with a lower density ^3^. However, excessive reduction of laser power or excessive increase of scanning speed results in extremely small nanostructures at very low densities, causing a lower absorption strength over the visible spectrum. In addition, the interline period adjusts the ratio between the laser-treated and untreated region, providing an additional degree of freedom to manipulate the absorption/emission spectra of the fs-laser processed solar absorbers.

The light absorber efficiency $\eta_{abs}$ at the given operating temperature $T_{abs}$ and solar concentration $C$, assuming only radiative losses, can be calculated using Equation 2 with a solar concentration of 1 and a solar intensity $I_{solar}=1000 W m^{-2}$. As shown in Figure 2d, a better SSA can be obtained using a lower laser power (25-50 mW) with a slower scanning speed (0.5-1 mm s^-1^) or using a higher laser power (250-500 mW) with a faster scanning speed (3-5 mm s^-1^). Both combinations of laser parameters could result in a relatively high solar absorption and low IR emission, leading to a high $\eta_{abs}$. Furthermore, for high laser powers, it is also possible to create good SSAs by increasing the ratio of untreated regions with a larger interline spacing. Since W is an intrinsic SSA, increasing the ratio of untreated region could still maintain a low average IR emissivity even though the fs-laser treated regions have a broadband absorption spectrum. The smallest interline spacings to obtain densely patterned fs laser treated W-SSAs are 40 μm, 50 μm, 80 μm and 90 μm for samples fabricated with laser power of 25 mW, 50 mW, 250 mW and 500 mW, respectively.

Note that by comparing the variation of absorption spectra in Figure S2c with Figure 2c, changes in laser scanning speed cause more change in absorption than laser power. This is because the Ti:sapphire fs laser used in this work has a low repetition rate of 1 kHz. By increasing laser scanning speed, there will be unirradiated areas between adjacent pulses, causing drastic decrease in solar absorption.


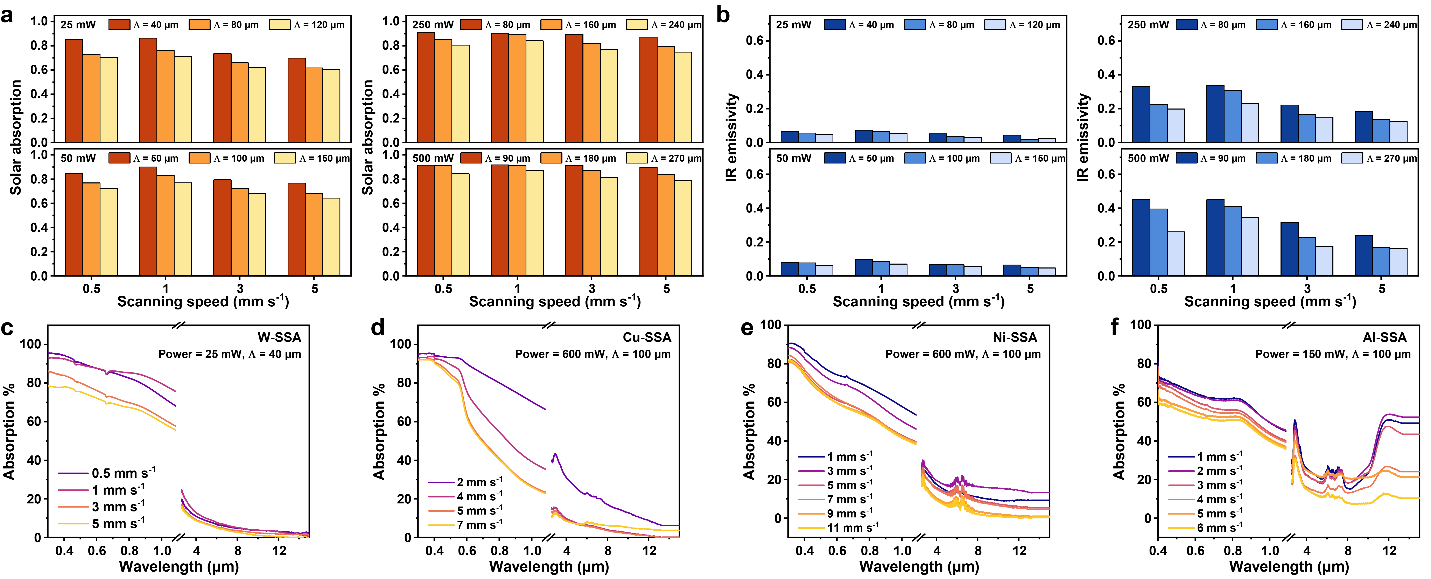


**Figure S2:** (a) Calculated solar absorptance $\bar{\alpha}$ and (b) Calculated IR emissivity $\bar{\varepsilon}$ for fs-laser treated W-SSAs with different laser processing parameters. (c-f) Relationship between spectral absorption and laser scanning speed for W-SSA (c), Cu-SSA (d), Ni-SSA (e), and Al-SSA (f).

**Supplementary Note 3: Optimization of greenhouse chamber**

In natural convection, air in contact with a hot surface absorbs heat, becomes less dense, and rises, allowing cooler, denser air to replace it. This process generates continuous air circulation that transfers heat away from the surface. However, when air is confined in the greenhouse chamber, circulation is restricted, leading to increased heat transfer through conduction. Due to air’s low thermal conductivity, it acts as an effective insulating material. The thickness of the air film is critical for optimal thermal insulation. If the film is too thin, insulation is limited because thermal conduction is inversely related to material thickness, while a film that is too thick permits more air circulation, reducing the insulation efficiency. To determine the ideal air film thickness, a physics interface of non-isothermal flow and conjugate heat transfer was carried out to study the air film insulation effect using COMSOL.

Laminar flow was used to model the natural convective air flow, and the governing equations are the Navier-Stokes equations expressed as

| $\rho\left( \boldsymbol{u}\cdot\nabla\right)\boldsymbol{u}=-\nabla p+\nabla\cdot\tau+\boldsymbol{F}+\rho g$ | (S3.1) |
| --- | --- |
| $\nabla\cdot\left( \rho\boldsymbol{u} \right)=0$ |  |

where $\rho$ is the density, $\boldsymbol{u}$ is the velocity vector, $p$ is the pressure, $\tau=\mu\left( \nabla\boldsymbol{u}+\left( \nabla\boldsymbol{u} \right)^{T} \right)-\frac{2}{3}\mu(\nabla\cdot\boldsymbol{u})\boldsymbol{I}$ is the viscous stress tensor with $\mu$ being the dynamic viscosity, $\boldsymbol{F}$ is the body force vector, and $g$ is the gravity. For the heat transfer in fluid, the governing equations are expressed as

| $\rho C_{p}\boldsymbol{u}\cdot\nabla T+\nabla\cdot\boldsymbol{q}=Q+Q_{p}+Q_{vd}$, $\boldsymbol{q}= -K\nabla T$ | (S3.2) |
| --- | --- |

where $\rho$ is the density, $C_{p}$ is the specific heat, $\boldsymbol{u}$ is the translation motion velocity, $T$is the temperature, $\boldsymbol{q}$ is the conduction heat flux, $Q$ is the heat source, $Q_{p}$ is the work done by pressure change, $Q_{vd}$ describes the viscous heating. The equations for heat transfer, and $K$ is the thermal conductance. The equations for heat transfer in solids are described in Equation S1.4.

The STEG hot side is simulated as a W absorber with a dimension of 20 mm × 20 mm × 0.2 mm, and 10 μm PE film elevated to a height $d$ above the absorber surface, as shown in Figure S3a. The vertical surfaces above the plastic film are defined as the air inlet, and the top surface is defined as the outlet. The air flow in and out of the computation space is driven by pressure. A small amount of heat loss through the supporting frames is also considered. A constant temperature heat source is defined at the absorber’s bottom surface, and its heating power is recorded as a measurement of thermal loss, as shown in Figure S3b. We evaluated the effectiveness of the insulation air film by comparing to the case without the plastic film

| $Heat loss reduction=\frac{P_{air pocket}(d)-P_{no air pocket}}{P_{no air pocket}}$ | (S3.3) |
| --- | --- |

where $P_{air pocket}(d$) is the heating power for the case with an air-film thickness of $d$, and $P_{no air pocket}$ is the heating power for the case without the air film. The results are shown in Figure 3d.

To verify the greenhouse chamber optimization result and its insulation effectiveness, we experimentally compared the heating power to maintain the absorber at a constant temperature with and without the greenhouse chamber. A joule heater was powered by a DC power source for heating, and the current (*I*) and voltage (*V*) across the heater was monitored using multimeters to calculate the heating power $P_{heater}$, as shown in Figure S3c. The surroundings and lower side of the heater were thermally insulated using ceramic fiber. The thermal loss of the air film is calculated as

| $P_{thermal loss}=P_{heater}-P_{loss}=\left( I\cdot V \right)-P_{loss}$ | (S3.4) |
| --- | --- |

where $P_{loss}$ was mainly due to thermal conduction through wires. $P_{loss}$ was measured by insulating both the upper and lower sides of the heater. Figure S3d shows the hot-side heat loss with different air-film thicknesses. The experimental result agrees well with the numerical modeling.


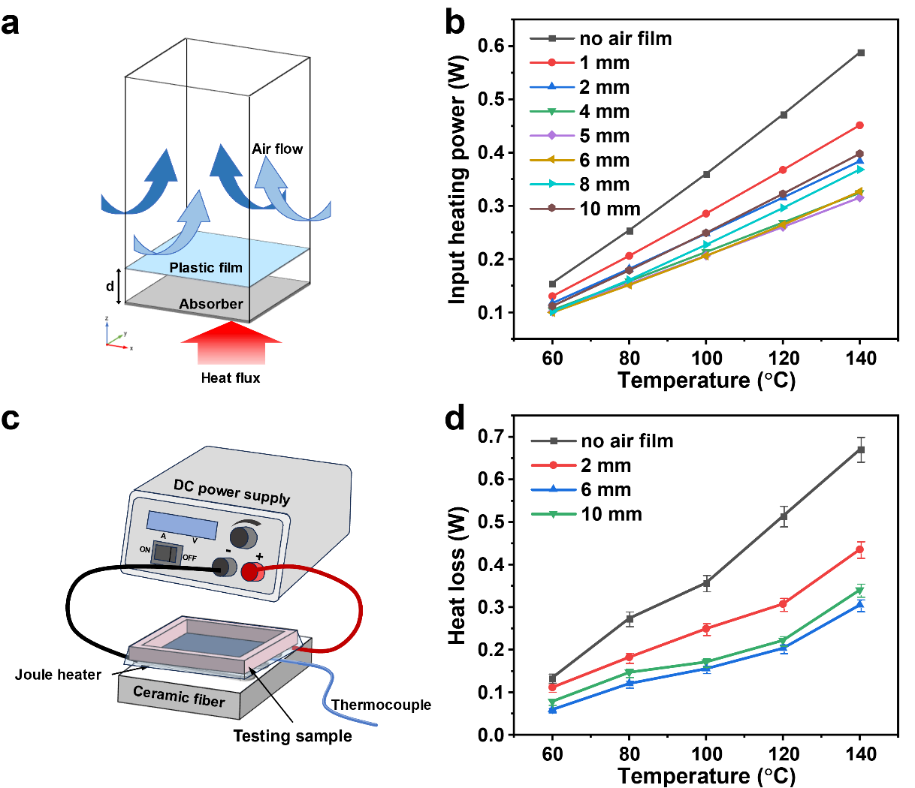


**Figure S3:** (a) Schematic of the greenhouse chamber simulation with various air-film thickness. (b) Simulated heat loss for different air-film thicknesses. (c) Schematic of the air film thermal loss measurement. (d) Measured heat loss for different air-film thicknesses.

**Supplementary Note 4: Micro thermal radiator’s (μ-dissipator) cooling power and weight**

The μ-dissipator’s cooling power is measured using the setup shown in Figure S4a. A joule heater is powered by a DC power source to heat the test sample, and the current (*I*) and voltage (*V*) across the heater is monitored using multimeters to calculate the cooling power $P_{cooling}=\left( I\cdot V \right)-P_{loss}$, where $P_{loss}$ is the thermal loss of the system (mainly due to thermal conduction through wires), and it is measured by insulating both the upper and lower sides of the joule heater. Meanwhile, the test sample’s temperature and the ambient temperature are monitored using thermocouples.

The μ-dissipator is created by removing material from the Al foil through fs-laser ablation. Besides enhancing cooling performance, the laser ablation process also reduces the mass of the heat dissipator. This reduction in weight could be especially important for portable or tracked systems ^4^. To take the heat dissipator’s weight into account, we can use the mass specific heat transfer coefficient to characterize its performance ^5^, which is defined as

|  | $h_{m}=\frac{Q}{m\cdot\left( T_{dissipator}-T_{amb} \right)}$ | (S4.1) |
| --- | --- | --- |

where $Q$ is the total heat dissipated through the dissipator, $m$ is the mass of the dissipator, $T_{dissipator}$ and $T_{amb}$ are respectively the temperature of the dissipator and the ambient. As shown in Figure S4b,c, we compare the weight and mass specific heat transfer coefficient of the optimized μ-dissipator, Al dissipator and a typical commercial parallel-fin heat sink. Commercial heat sinks with parallel-fin structures are widely used for passive cooling systems. We use a commercial heat sink that has the same cross section area as the μ-dissipator and Al dissipator, and it has a cubic shape (15 mm × 15 mm × 15 mm). Although the commercial heat sink has higher absolute cooling power due to its larger size, it is substantially heavier than the μ-dissipator. Under the same temperature, the mass specific heat transfer coefficient of the μ-dissipator is ~210% of an Al dissipator and more than 670% of a typical commercial heat sink, suggesting a superior heat dissipation efficiency of the μ-dissipator while maintaining a light weight. In another word, μ-dissipator only needs about 15% of the material to achieve the same cooling performance as a commercial heat sink, which can greatly reduce the material cost. Additionally, unlike a bulky regular heat sink that may not fit devices with complex geometries, the μ-dissipator can be directly fabricated on the device’s outer shell using fs-laser processing. This allows more flexibility in thermal management.


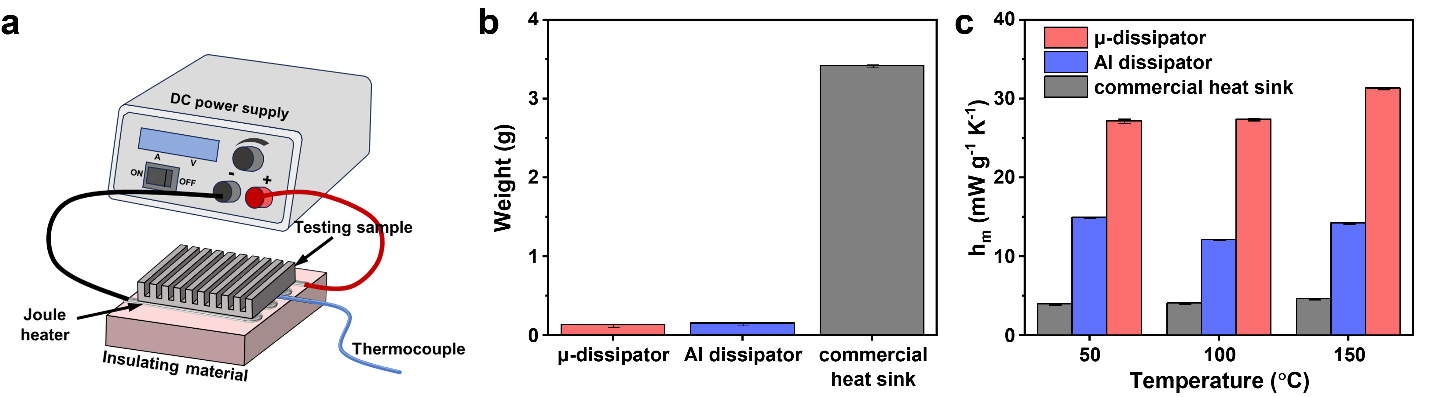


**Figure S4.** (a) Schematic of the cooling power measurement. The test sample is heated by a joule heater, and the temperature and heating power are recorded to calculate the cooling power. (b) Weight and (c) mass specific heat transfer coefficient of the optimized μ-dissipator, Al dissipator and a typical commercial parallel-fin heat sink.

**Supplementary Note 5: Optimization of micro thermal radiator (****μ-dissipator)**

The fs-laser-induced surface structures affect not only the convective cooling but also the radiative cooling of the μ-dissipator. Compared with untreated metal surface, convective cooling of the μ-dissipator was enhanced by increasing the surface area. A μ-dissipator with deeper and narrower microgrooves could have a larger surface area. However, air flow inside the deep and narrow grooves may be stagnated due to air viscosity, causing a lower heat transfer efficiency ^6, 7^. To find the optimal μ-dissipator surface topography, a numerical simulation of non-isothermal flow and conjugate heat transfer using COMSOL was carried out. The physics interfaces involved in modeling are similar to Supplementary Note 3.

As it’s time-consuming to model the fs-laser induced irregular surface microstructures, we approximate the laser-treated Al surface as rectangular grooves with different groove width and depth, as shown in Figure S5a. The grooves are densely packed on an Al substrate with a dimension of 20 mm × 20 mm × 0.2 mm. The vertical surfaces are defined as the air inlet, and the top surface is defined as the outlet. A 100 ℃ constant temperature heat source is defined at the bottom surface, and the heating power is recorded. We evaluated the μ-dissipator’s convective cooling capacity by comparing it to a flat Al surface.

| $Convective cooling enhancement=\frac{P_{conv,\mu-HSp}(H,S)-P_{conv,Al HSp}}{P_{conv,Al HSp}}$ | (S5.1) |
| --- | --- |

where $P_{conv, \mu-HSp}(H,S)$ is the convective cooling power for a μ-dissipator with grooves that have a depth of $H$ and a width of $S$, and $P_{conv,Al HSp}$ is the convective cooling power for a flat Al surface. The results are shown in Figure 4c.

The IR emissivity of the μ-dissipators with different surface topography was measured (representative samples shown in Figure S5b,c), and an emissivity map was obtained by curve fitting of the experimental data points up to the third order, as shown in Figure S5d. The variation of IR emissivity with the surface geometries is because different laser parameters were required to obtain these surface geometries, which alter the micro- and nano-scale surface structures and hence affects the IR emissivity. To evaluate the μ-dissipator’s radiative cooling enhancement, we compared the calculated radiative cooling power at 100 ℃ with the Al Dissipator made of flat Al surface.

| $Radiative cooling enhancement=\frac{P_{rad, \mu-HSp}(H,S)-P_{rad,Al HSp}}{P_{rad, Al HSp}}$ | (S5.2) |
| --- | --- |

where $P_{rad,\mu-HSp}(H,S)$ is the radiative cooling power for a μ-dissipator with grooves that have a depth of $H$ and a width of $S$, and $P_{rad,Al HSp}$ is the convective cooling power for a flat Al surface. The results are shown in Figure 4d. And the combined enhancement of cold-side cooling power through convection and radiation is calculated as

| $Cold-side cooling enhancement=\frac{[P_{conv,\mu-HSp}\left( H,S \right)+P_{rad, \mu-HSp}\left( H,S \right)]-(P_{conv,Al HSp}+P_{rad,Al HSp})}{P_{conv,Al HSp}+P_{rad,Al HSp}}$ | (S5.3) |
| --- | --- |

And the results are shown in Figure 4e.

The cooling power enhancement of the optimized μ-dissipator is experimentally verified using setup shown in Figure S4a. The cooling power for μ-dissipator and Al dissipator at 50, 100, and 150 ℃ is measured and shown in Figure S5e. At 100 ℃, μ-dissipator shows a 1.9× enhancement in cooling power.


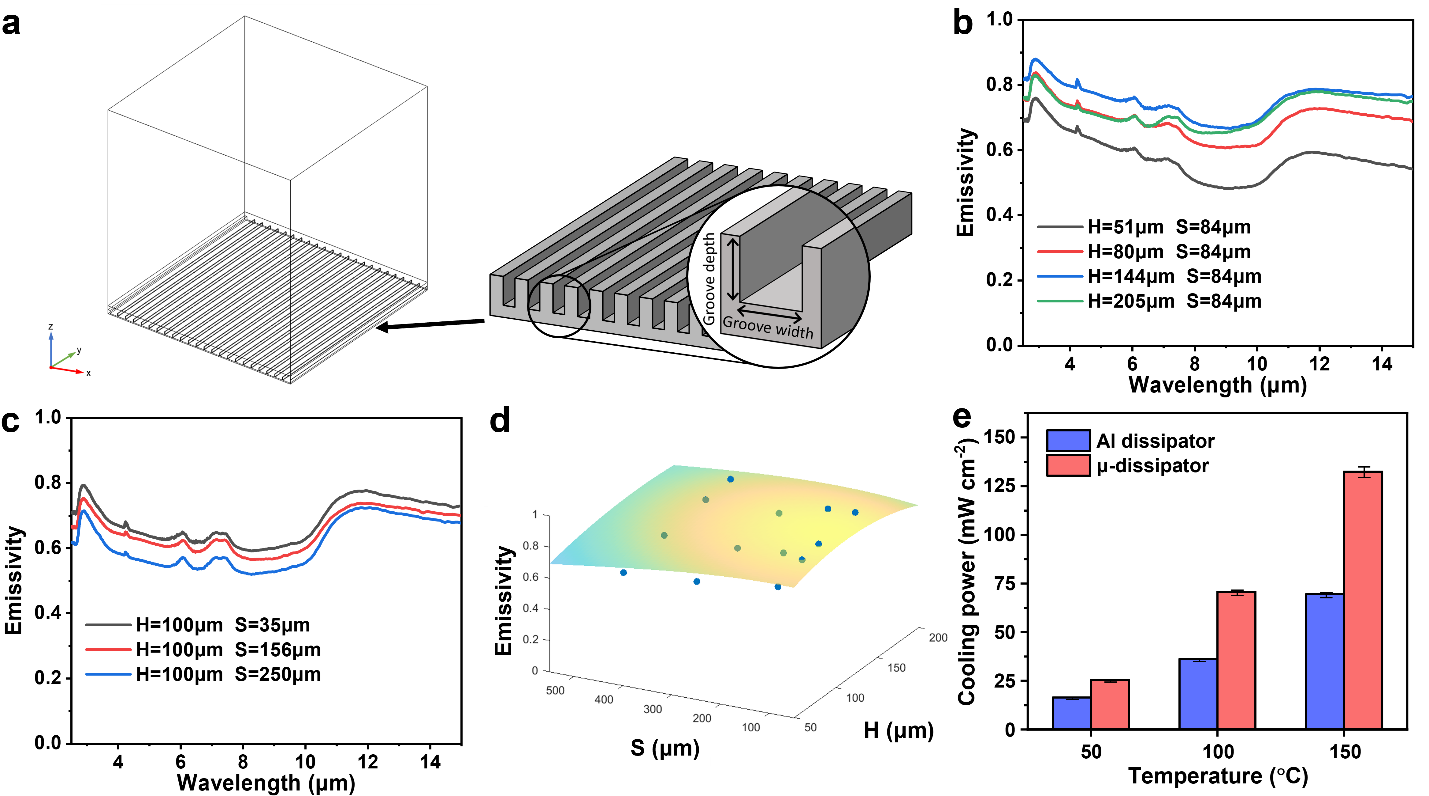


**Figure S5.** (a) Geometry of the μ-dissipator for numerical modeling. (b) Spectral emissivity of μ-dissipator samples with various groove depth and a groove width of 84 μm. (c) Spectral emissivity of μ-dissipator samples with various groove width and a groove depth of 100 μm. (d) IR emissivity map fitted by experimental data. (e) Cooling power measurements for μ-dissipator and Al dissipator at 50, 100, and 150 ℃.

**Supplementary Note 6: Thermal insulation effect of air film**

The absorber temperatures of three different setups were measured: solar absorber without polyethylene (PE) film, directly covered by PE film, and covered by PE film with an air film (Figure S6a). Figure S6b shows the transmission and reflection spectrum of the PE film. A solar radiation of approximately 1 sun (1000 W m^-2^) was applied to the absorber. The lower side of the solar absorber was thermally insulated using insulating foam. The thermal insulation capacity of air film was measured by comparing the absorber temperature in the three cases. The solar absorber directly covered by PE film has a slightly lower steady state temperature compared to absorber without plastic film (Figure S6c). Although the PE film has a low thermal conductivity, the absorber directly covered by PE film reached a temperature that is ~98% of the that without PE film, indicating that the thermal insulating effect of the PE film cannot compensate for the slight optical loss due to its thin thickness. For the absorber covered by PE film with an air film, the convection loss was effectively reduced, and ~15% increase in absorber temperature was achieved, suggesting that the contribution of thermal insulation was mainly from the air film.


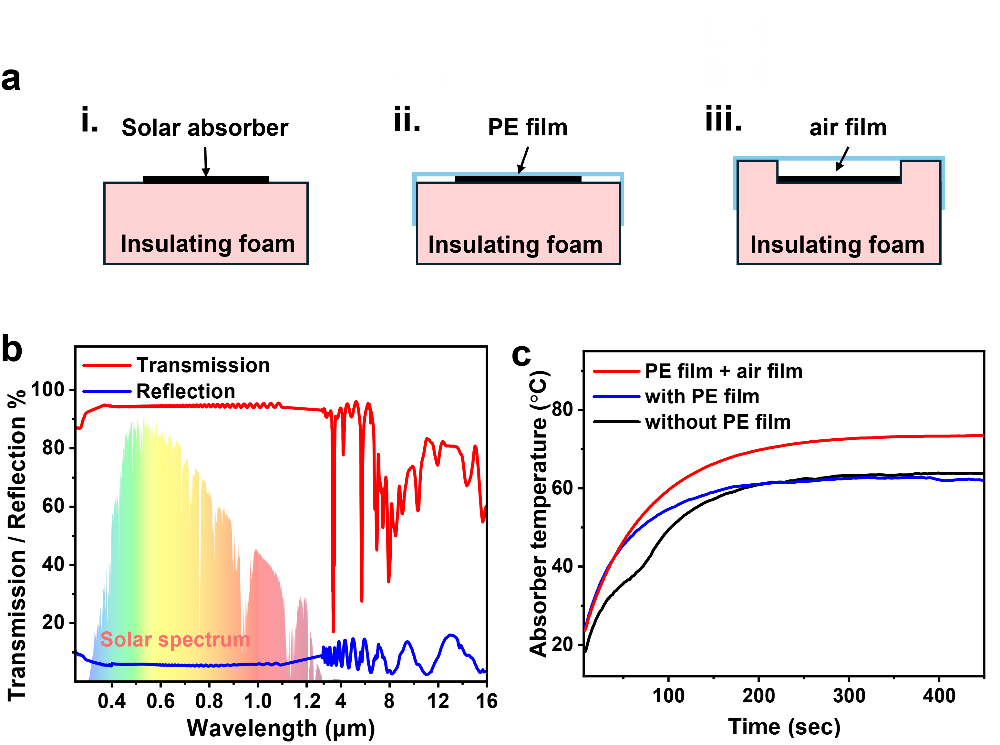


**Figure S6.** (a) Schematics of the three testing setups: (i) solar absorber without PE film, (ii) directly covered by PE film, and (iii) covered by PE film with an air film. (b) Transmission and reflection spectrum of the PE film. (c) The solar absorber temperature with respect to time.

**Supplementary Note 7: Durability and environmental analysis of the STEG**

In real-world applications, the durability of STEG in outdoor environments is a great concern, especially the greenhouse chamber since it is the outmost shell of the STEG. To test the durability of the greenhouse chamber under extensive outdoor use, we perform a series of stress tests, e.g., high temperature and high wind speed.

To test its stability under high temperatures, we put the greenhouse chamber on a hot plate and gradually raise the temperature until the plastic film melts, as shown in Figure S7a. To withstand the high temperature of the hot plate, we use insulating material with a high melting point to make the frame to support the plastic film, which directly contacts the hot plate, and the overall structure of the greenhouse chamber maintains the same. The greenhouse chamber has an air-film thickness of ~6 mm. We raise the hot plate temperature from ~150 ℃ with an increment ~50 ℃. Figure S7b shows the temperature of the hot plate and the plastic film. Due to the low air conduction and convection in the greenhouse chamber, the temperature of the plastic film is much lower than the hot plate. The plastic film starts to melt at ~150 ℃. At this moment, the hot plate temperature is over 320 ℃, which is 200 ℃ higher than the temperature limit of the tested TEG (~120 ℃). Since the elevated plastic film can easily withstand a hot surface temperature over 300 ℃, we believe the greenhouse chamber can survive in environments with extreme temperatures.

Besides high temperature, wind is also a concern for outdoor use. To test the greenhouse chamber’s durability under high wind blowing, we put the greenhouse chamber in front of a compressed air blower and blow at full power, as shown in Figure S7c. The compressed air blower can blow at 28 m s^-1^, which is equivalent to a Beaufort scale 10 storm. Under the destructive high wind speed, the greenhouse chamber stays intact, demonstrating its structural strength and stability in real-world applications.

Furthermore, we also investigate the influence of wind on STEG power generation. The STEG with and without thermal management is placed under a solar simulator, and an electric fan blows constant wind from the side, as shown in Figure S7d. At the same time, to demonstrate the scalability of spectral engineering and thermal management on STEG, we utilize a TEG with larger size (30 mm × 30 mm, TE Technology). This STEG is 2.25 times larger than the one we used in the main text. Figure S7e,f shows the STEG power under wind speeds of 0 m s^-1^, 1 m s^-1^, and 2 m s^-1^ with and without thermal management respectively. For STEG without thermal management, though air flow increases the convective cooling on the cold side, the convective loss on the hot side also increases. As a result, the overall ΔT across the TEG decreases, and STEG power drops at higher wind speeds. In contrast, STEG with spectral and thermal management on both sides generates more power at higher wind speed. This is because the STEG hot side is covered by the greenhouse chamber, and the hot side convective loss is minimized. The air flow not only enhances the cold-side convective cooling, increasing the ΔT across the TEG, but also lowers the STEG average temperature, increasing the TEG efficiency. Therefore, wind has a positive effect on STEG with the greenhouse chamber and further enhances STEG power generation.


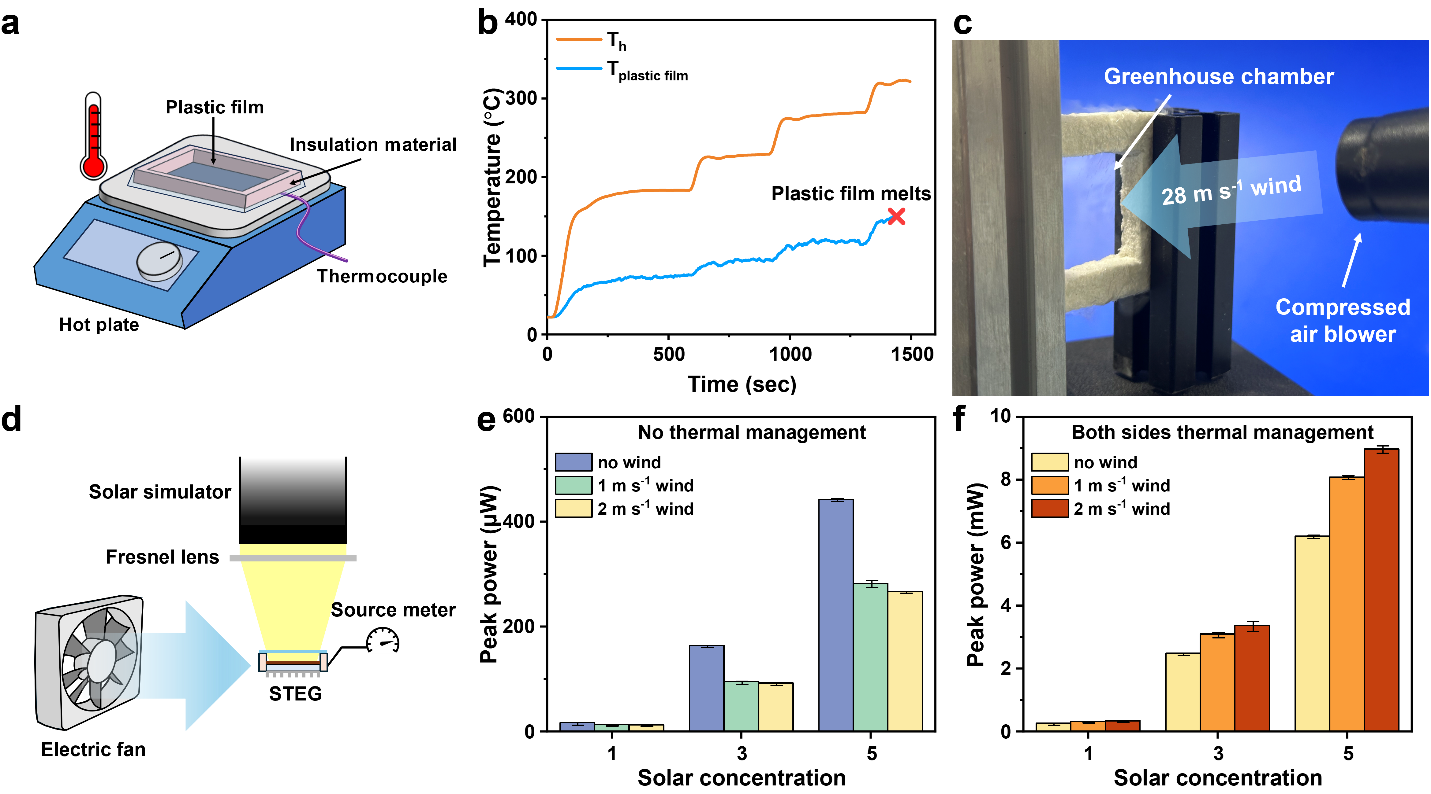


**Figure S7.** (a) Schematic of stress test for the greenhouse chamber under high temperatures. (b) Temperatures of the greenhouse chamber plastic film and the hot plate surface. The plastic film starts to melt at ~150 ℃. At this moment, the hot plate temperature is over 320 ℃. (c) Photo of stress test for the greenhouse chamber under high-speed wind blowing. The greenhouse chamber stays intact under a 28 m s^-1^ wind. (d) Schematic of STEG performance test under various wind speed. (e) STEG power generation under wind speeds of 0 m s^-1^, 1 m s^-1^, and 2 m s^-1^ without thermal management. (f) STEG power generation under wind speeds of 0 m s^-1^, 1 m s^-1^, and 2 m s^-1^ with thermal management on both sides.

**Supplementary Note 8: Efficiency analysis of the STEG**

STEG efficiency can be calculated using the following equation:

| $\eta=\frac{P_{STEG}}{P_{in}}$ | (S8.1) |
| --- | --- |

where $P_{STGE}$ is the maximum power output of the STEG, and $P_{in}$ is the input solar energy. Figure S8a shows the efficiency of the STEG with and without the spectral and thermal management at different solar concentrations. Note that due to the limited power and aperture size of our solar simulator, we have to use a TEG with low thermoelectric efficiency and high internal resistance to match the dimension requirement at solar concentrations > 5 suns. As the solar concentration increases, the efficiency of the STEG with both-side spectral and thermal management increases, while the efficiency of the STEG without thermal management almost stays constant. This indicates that the spectral and thermal management has a better performance at higher temperatures. Our work focuses on STEG power enhancement rather than absolute efficiency. In fact, even using a low-cost and low-efficiency TEG, our spectral and thermal management can make it usable in STEG applications, such as powering LEDs (Figure 5f).

Moreover, the low absolute efficiency of the STEG device can be resolved by utilizing TE materials with higher thermoelectric efficiencies. As demonstrated in the scalability test of the STEG device, a TEG with higher thermoelectric efficiency is used (Supplementary Note 7). Figure S8b shows the efficiency and power enhancement of the STEG with and without the spectral and thermal management at different solar concentrations. At 5 suns, STEG with spectral and thermal management achieves an efficiency of ~0.14%, and it has the same extent of power enhancement compared to the case of low-efficiency TEG. Therefore, STEG power enhancement through our spectral engineering and thermal management strategy is independent of the TEG efficiency. With the advancement of high-efficiency TE materials, we believe our spectral engineering and thermal management can expand the applications of STEG devices.


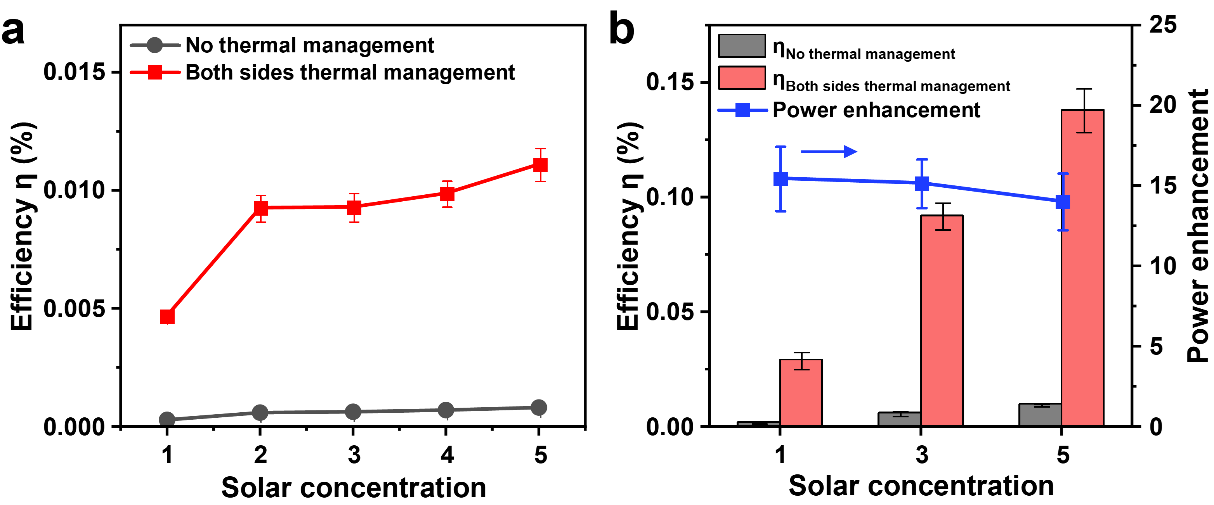


**Figure S8.**  (a) Efficiency of the low-cost and low-efficiency STEG (20 mm × 20 mm) with and without the spectral and thermal management at different solar concentrations. (b) Efficiency and power enhancement of the high-efficiency STEG (30 mm × 30 mm) with and without the spectral and thermal management at different solar concentrations.

**Supplementary Note 9: Scalability and environmental impact of fs laser processing**

As a powerful processing tool, fs laser direct writing has shown its advantages in high spatial resolution, high accuracy, 3D processing capability, and a wide range of processable materials in the field of scientific research ^8^. However, the equipment financial cost, processing time, and energy consumption is more concerned for industrial-scale fabrication.

The femtosecond laser used in this work is a Ti:sapphire high-intensity laser with a maximum average power of 7 W and a repetition rate of 1 kHz, and the scanning speed was limited by the low repetition rate. For industrial scaling, femtosecond laser system providing higher power and faster repetition rate can be used. Correspondingly, a faster scanning speed is enabled with reduced fabrication time as well as the cost. In this work, a conventional single beam processing is used, and the laser processing time can be further shortened by applying multibeam parallel processing ^9^. Using a spatial light modulator (SLM), the input beam can be modulated to generate multiple parallel beams ^10^, and massively parallel fs laser processing with more than 1000 beams has been demonstrated ^11^. This technique greatly enhanced the throughput of fs laser processing. Moreover, unlike common fabrication methods such as E-beam lithography and photolithography, fs laser processing does not involve sophisticated material preparation and development steps, which shortens the setup time. A standard lab cleaning procedure will be sufficient for materials preparation in fs laser processing.

At present, as compared with E-beam lithography and photolithography, the use of fs laser processing for industrial-scale fabrication is still at an early stage. The high initial investment of the fs laser may hinder its application in industry. However, compared to photolithography, fs laser processing is a simple, single-step fabrication method and does not require extra steps such as photomask fabrication and etching, which requires additional equipment. Also, fs lasers do not require clean room environments for operation, which further reduces its operation cost.

The power consumption of fs laser varies with models and average output power. A typical industrial fs laser with maximum output power of 20 W has only 600 W electric power consumption (Light Conversion, Pharos PH2-UP), which is equivalent to kitchen appliances. More powerful fs lasers with maximum output of 120 W can consume 1500 W electric power (Light Conversion, Carbide CB3-120W), which is still in an affordable range for industrial applications. While the power consumption of chillers may be higher than the laser system, the waste heat can be used to generate electricity using thermoelectric waste heat recovery systems ^12^ or be utilized by thermal storage systems ^13^. Applying waste heat recovery to the cooling system can substantially reduce energy waste during fs laser processing.

To date, fs lasers have been widely studied in precision engineering, enabling feature sizes ranging from the micron to nanoscale. Despite its many advantages, there are still challenges that need to be overcome. For instance, there is a need to develop fs laser precision nano structuring in the far-field and ambient air since near-field processing is restricted by the short working distance ^9^. In far-field operation, the achievable feature size remains constrained by the optical diffraction limit. Potential solutions include performing fs laser processing in water ^14^, and performing double-pulse irradiation ^15^.

**References**

1. Prasad, A. and R.C. Thiagarajan. *Multiphysics modeling and multilevel optimization of thermoelectric generator for waste heat recovery*. in *Proceedings of the COMSOL Conference*. 2018.

2. Jaziri, N., et al., *A comprehensive review of Thermoelectric Generators: Technologies and common applications.* Energy reports, 2020. **6**: p. 264-287.

3. Jalil, S.A., et al., *Spectral absorption control of femtosecond laser-treated metals and application in solar-thermal devices.* Light: Science & Applications, 2020. **9**(1): p. 14.

4. Micheli, L., S. Senthilarasu, K. Reddy, and T.K. Mallick, *Applicability of silicon micro-finned heat sinks for 500× concentrating photovoltaics systems.* Journal of Materials Science, 2015. **50**: p. 5378-5388.

5. Bar-Cohen, A., M. Iyengar, and A.D. Kraus, *Design of optimum plate-fin natural convective heat sinks.* J. Electron. Packag., 2003. **125**(2): p. 208-216.

6. Micheli, L., K. Reddy, and T.K. Mallick, *General correlations among geometry, orientation and thermal performance of natural convective micro-finned heat sinks.* International journal of heat and mass transfer, 2015. **91**: p. 711-724.

7. Mahmoud, S., et al., *Effect of micro fin geometry on natural convection heat transfer of horizontal microstructures.* Applied Thermal Engineering, 2011. **31**(5): p. 627-633.

8. Xu, B.-B., et al., *Fabrication and multifunction integration of microfluidic chips by femtosecond laser direct writing.* Lab on a Chip, 2013. **13**(9): p. 1677-1690.

9. Lin, Z. and M. Hong, *Femtosecond laser precision engineering: from micron, submicron, to nanoscale.* Ultrafast Science, 2021.

10. Lei, P., et al., *Femtosecond laser multibeam parallel processing for variable focal-length optofluidic chips.* Optics Letters, 2023. **48**(21): p. 5603-5606.

11. Hasegawa, S., H. Ito, H. Toyoda, and Y. Hayasaki, *Massively parallel femtosecond laser processing.* Optics express, 2016. **24**(16): p. 18513-18524.

12. Bell, L.E., *Cooling, heating, generating power, and recovering waste heat with thermoelectric systems.* science, 2008. **321**(5895): p. 1457-1461.

13. Du, K., J. Calautit, P. Eames, and Y. Wu, *A state-of-the-art review of the application of phase change materials (PCM) in Mobilized-Thermal Energy Storage (M-TES) for recovering low-temperature industrial waste heat (IWH) for distributed heat supply.* Renewable Energy, 2021. **168**: p. 1040-1057.

14. Liu, J., et al., *Direct writing of 150 nm gratings and squares on ZnO crystal in water by using 800 nm femtosecond laser.* Optics Express, 2014. **22**(26): p. 32361-32370.

15. Mastellone, M., et al., *Deep-subwavelength 2D periodic surface nanostructures on diamond by double-pulse femtosecond laser irradiation.* Nano Letters, 2021. **21**(10): p. 4477-4483.
